# *De novo* variants of *CSNK2B* cause a new intellectual disability-craniodigital syndrome by disrupting the canonical Wnt signaling pathway

**DOI:** 10.1016/j.xhgg.2022.100111

**Published:** 2022-04-18

**Authors:** Maria Asif, Emrah Kaygusuz, Marwan Shinawi, Anna Nickelsen, Tzung-Chien Hsieh, Prerana Wagle, Birgit S. Budde, Jennifer Hochscherf, Uzma Abdullah, Stefan Höning, Christian Nienberg, Dirk Lindenblatt, Angelika A. Noegel, Janine Altmüller, Holger Thiele, Susanne Motameny, Nicole Fleischer, Idan Segal, Lynn Pais, Sigrid Tinschert, Nadra Nasser Samra, Juliann M. Savatt, Natasha L. Rudy, Chiara De Luca, Italian Undiagnosed Diseases Network, Paola Fortugno, Susan M. White, Peter Krawitz, Anna C.E. Hurst, Karsten Niefind, Joachim Jose, Francesco Brancati, Peter Nürnberg, Muhammad Sajid Hussain

**Affiliations:** 1 Cologne Center for Genomics (CCG), University of Cologne, Faculty of Medicine and University Hospital Cologne, 50931 Cologne, Germany; 2 Center for Biochemistry, Medical Faculty, University of Cologne, 50931 Cologne, Germany; 3 Center for Molecular Medicine Cologne (CMMC), University of Cologne, Faculty of Medicine and University Hospital Cologne, 50931 Cologne, Germany; 4 Bilecik Şeyh Edebali University, Molecular Biology and Genetics, Gülümbe Campus, 11230 Bilecik, Turkey; 5 Division of Genetics and Genomic Medicine, Department of Pediatrics, Washington University School of Medicine, St. Louis, MO, USA; 6 Institute of Pharmaceutical and Medicinal Chemistry, Westphalian Wilhelms-University, Münster, Germany; 7 Institute for Genomic Statistics and Bioinformatics, University Hospital Bonn, Rheinische Friedrich Wilhelms, Universität Bonn, Bonn, Germany; 8 Cologne Excellence Cluster on Cellular Stress Responses in Aging-Associated Diseases (CECAD), University of Cologne, Cologne, Germany; 9 Department of Chemistry, Institute of Biochemistry, University of Cologne, Cologne, Germany; 10 University Institute of Biochemistry and Biotechnology (UIBB), PMAS-Arid Agriculture University, Rawalpindi, Pakistan; 11 Berlin Institute of Health at Charité – Universitätsmedizin Berlin, Core Facility Genomics, Charitéplatz 1, 10117 Berlin, Germany; 12 Max Delbrück Center for Molecular Medicine in the Helmholtz Association (MDC), Berlin, Germany; 13 FDNA Inc., Boston, MA, USA; 14 Hospital Center, Safed, Israel; 15 Center for Mendelian Genomics, Broad Institute of MIT and Harvard, Cambridge, MA, USA; 16 Zentrum Medizinische Genetik, Medizinische Universität, Innsbruck, Austria; 17 Azrieli Faculty of Medicine, Bar-Ilan University, Safed, Israel; 18 Genomic Medicine Institute, Geisinger, Danville, PA, USA; 19 Department of Genetics, University of Alabama at Birmingham, Birmingham, AL, USA; 20 Department of Life, Health and Environmental Science, University of L’Aquila, 67100 L’Aquila, Italy; 21 IRCCS, San Raffaele Roma, 00163 Roma, Italy; 22 Victorian Clinical Genetics Services, Murdoch Children’s Research Institute, Melbourne, VIC, Australia; 23 Department of Paediatrics, University of Melbourne, Melbourne, VIC, Australia

**Keywords:** intellectual disability-craniodigital syndrome, *CSNK2B*, CK2β, CK2, Wnt signaling, β-catenin, DVL3, CK2α, GestaltMatcher, POBINDS, whole transcriptome profiling, whole-phosphoproteome profiling

## Abstract

*CSNK2B* encodes for casein kinase II subunit beta (CK2β), the regulatory subunit of casein kinase II (CK2), which is known to mediate diverse cellular pathways. Variants in this gene have been recently identified as a cause of Poirier-Bienvenu neurodevelopmental syndrome (POBINDS), but functional evidence is sparse. Here, we report five unrelated individuals: two of them manifesting POBINDS, while three are identified to segregate a new intellectual disability-craniodigital syndrome (IDCS), distinct from POBINDS. The three IDCS individuals carried two different *de novo* missense variants affecting the same codon of *CSNK2B*. Both variants, NP_001311.3; p.Asp32His and NP_001311.3; p.Asp32Asn, lead to an upregulation of *CSNK2B* expression at transcript and protein level, along with global dysregulation of canonical Wnt signaling. We found impaired interaction of the two key players DVL3 and β-catenin with mutated CK2β. The variants compromise the kinase activity of CK2 as evident by a marked reduction of phosphorylated β-catenin and consequent absence of active β-catenin inside nuclei of the patient-derived lymphoblastoid cell lines (LCLs). In line with these findings, whole-transcriptome profiling of patient-derived LCLs harboring the NP_001311.3; p.Asp32His variant confirmed a marked difference in expression of genes involved in the Wnt signaling pathway. In addition, whole-phosphoproteome analysis of the LCLs of the same subject showed absence of phosphorylation for 313 putative CK2 substrates, enriched in the regulation of nuclear β-catenin and transcription of the target genes. Our findings suggest that discrete variants in *CSNK2B* cause dominant-negative perturbation of the canonical Wnt signaling pathway, leading to a new craniodigital syndrome distinguishable from POBINDS.

## Introduction

Intellectual disability-craniodigital syndrome (IDCS) refers to cranial anomalies, including microcephaly, facial dysmorphism, and digital anomalies of upper and/or lower limbs (syndactyly, brachydactyly, polydactyly, hyperphalangism, and clinodactyly). Neurologically, IDCS presents intellectual disability and epilepsy.^1^ IDCS is an umbrella term, lumping together different conditions with overlapping clinical features. Of these, Filippi syndrome (MIM: 272440), Chitayat syndrome (MIM: 617180), and Jawad syndrome (MIM: 251255) are conditions with identified genetic causes, whereas genetic underpinnings of Woods syndrome (MIM: 615236) are underexplored.^2^, ^3^, ^4^, ^5^

Pathogenic *CSNK2B* (MIM: 115441) variants have been reported to co-segregate with global developmental delay and epilepsy, a condition termed Poirier-Bienvenu neurodevelopmental syndrome (POBINDS; MIM: 618732).^6^, ^7^, ^8^ Experiments on the functional consequences of the *CSNK2B* variants were not presented in any of these reports.

The protein kinase casein kinase II (CK2) is an ubiquitously expressed Ser/Thr kinase with a repertoire of more than 450 physiological substrates.^9^ The holoenzyme is a heterotetramer of two α subunits structured around the obligate β dimer with three possible combinations: αββα, αββα′, or α′ββα′.^10^ The two catalytic subunits, α and α′, are encoded by the genes *CSNK2A1* (MIM: 115440) and *CSNK2A2* (MIM: 115442), respectively, whereas the regulatory subunit (CK2 subunit beta [CK2β]) is encoded by *CSNK2B*.^10^ These subunits may also exist in isolation and are supposed to have holoenzyme-independent roles in the cell as well.^11^ One of the reported facilitative roles of CK2β within the CK2 holoenzyme is substrate docking or recruitment; CK2β contributes to the recognition of the target substrate and promotes in this way the phosphorylation reaction.^12^ Generally, the catalytic activity of CK2α is switched neither on nor off on holoenzyme formation; nonetheless, phosphorylation of particular substrates can be deeply altered in this way.^13^ These substrates are broadly classified in three categories: class I substrates can be phosphorylated either by the holoenzyme or the catalytic subunits; class II substrates can be phosphorylated only by the catalytic subunits, and here CK2β plays an inhibitory role; and class III substrates can be phosphorylated only by the holoenzyme, which means that the substrates must be recognized and docked by the regulatory subunit.^13^

The role of CK2 is remarkably established in numerous important cellular activities, such as cell proliferation, differentiation, apoptosis, and DNA repair. CK2 positively regulates the Wnt signaling pathway, a key signaling event for normal embryogenesis. The associated modulation of gene expression is initiated by binding of Wnt proteins to the cell surface Frizzled receptors, which results in an inhibition of glycogen synthase kinase-3β (GSK-3β), a kinase that phosphorylates β-catenin.^14^ On inhibition of GSK-3β, β-catenin accumulates in the cytoplasm, forms a complex with DVL3, and translocates into the nucleus, where it binds to a family of DNA-binding proteins known as lymphoid enhancer-binding factor and T cell factor (LEF-1/TCF).^15^ CK2 phosphorylates Lef-1 at Ser40 and β-catenin at Thr393 located in the armadillo (arm) repeat domain necessary to interact with Lef-1.^14^^,^^16^^,^^17^ On phosphorylation of LEF-1 by CK2, its binding affinity for β-catenin is increased, and the affinity for transducin-like enhancer protein 1 (TLE1), which is a negative regulator of Wnt signaling, is decreased.^17^

CK2β is known to play a crucial role in the development of the central nervous system and organogenesis. Loss of *Csnk2b* in embryonic neural stem cells compromises proliferation and differentiation of embryonic neural stem/progenitor cells and oligodendrogenesis in the mouse telencephalon.^18^ Knockout of *Csnk2b* in mice leads to post-implantation lethality. Embryos showed a reduced size at embryonic day (E) 6.5 and were resorbed at E7.5. Homozygous *Csnk2b* knockout morula did not further develop after the blastocyst phase *in vitro*. A conditional knockout study revealed that lack of *Csnk2b* is deleterious for mouse embryonic stem cells (ESCs) and primary embryonic fibroblasts.^19^

Here, we report five patients carrying suspected *de novo* variants of *CSNK2B*, and we propose a novel IDCS in three of them based on computer-assisted differential diagnosis. We have also investigated the consequences of the underlying missense variants on structure and function of CK2β and show that impaired Wnt signaling causes the observed new phenotype.

## Material and methods

### Subjects

Through international collaborators and GeneMatcher,^20^ we recruited three affected members (subjects 1–3) manifesting a new IDCS, and two subjects (4 and 5) were clinically diagnosed with POBINDS. Notably, subject 5 was recruited through GenomeConnect, the ClinGen patient registry.^21^ We obtained written informed consents from the parents of the affected individuals. The protocol of this study on human material was approved by the Institutional Review Boards of the University of Cologne, Faculty of Medicine and University Hospital Cologne, Germany and University of L’Aquila (Italian Undiagnosed Rare Diseases Network), Italy.

### GestaltMatcher analysis

We applied GestaltMatcher^22^ to compare facial phenotypes among individuals with variants in *CSNK2B*. The detailed method of GestaltMatcher is provided in the extended supplemental material and methods. Briefly, we performed pairwise comparisons on the seven photos (subjects 1–7); this includes two subjects, 6 and 7, from the literature as well,^8^^,^^23^^,^^24^ together with 3,533 images from 2,516 diagnosed patients manifesting 816 syndromes enlisted in the Face2Gene (FDNA, USA) database.

### Whole-exome sequencing

To reveal the disease-causing DNA variant(s), we subjected either trios, subject and both parents, or only affected members for exome sequencing. The detailed procedure is given in the supplemental material and methods section.

### Copy number analysis

We subjected the DNA of subject 1 for copy number analyses. The procedure is given in the supplemental information.

### *In silico* methods

We predicted the pathogenicity of *CSNK2B* variants through multiple *in silico* tools (Table S1). For estimation of evolutionary conservation of mutated residues, we aligned the reference sequences of the orthologs retrieved from UniProtKB and/or NCBI using Clustal Omega (EMBL-EBI, Wellcome Trust Genome Campus, Hinxton, Cambridge, UK).

To rationalize the potential impact of variants at CK2β position 32 and to visualize this position within the CK2α_2_β_2_-holoenzyme assembly, we constructed a suitable functional complex by *in silico* 3D modeling. To this end, we loaded the CK2α_2_β_2_-holoenzyme structure with PDB: 1JWH^10^ into crystallographic object-oriented toolkit (COOT).^25^ Then, using the structural superimposition option of COOT, the two catalytic subunits were exchanged with a human CK2α structure in complex with two sulfate ions (PDB: 2PVR).^26^ These sulfate ions served as fixed points to place the p+1 and p+3 side chains of a substrate peptide (sequence DDSDDD) that was modeled into the active site as described by Niefind et al.^26^ The ATP analogue adenosine-5’-(β,γ-imido)triphosphate (AMPNP) present in PDB: 2PVR was deleted because it is disordered in the γ-phospho group region; instead, AMPPNP in complex with two magnesium ions was taken over from the maize CK2α structure with PDB: 1LP4,^27^ where it is well defined, after structural overlay of the protein matrices in COOT.^25^ Finally, the two critical CK2β variants, either NP_001311.3;Asp32Asn or NP_001311.3;Asp32His, were introduced with the amino acid exchange option of COOT;^25^ in this context, two preferred side-chain conformations of histidine and asparagine according to the COOT-internal rotamer database were selected.

### Minigene construction, transfection, and RT-PCR

The detailed procedure is provided in the supplemental material and methods.

### Cell culture

We generated lymphoblastoid cell lines (LCLs) from subjects 1 and 2 from blood samples collected in lithium heparin-coated tubes (BD Vacutainer PST). Red blood cells were lysed through incubation in lysis buffer (155 mM ammonium chloride, 10 mM potassium hydrogen carbonate, and 0.1 mM disodium-EDTA) for 10 min. Pelleted lymphocytes were immortalized by transfection of Epstein-Barr virus, followed by incubation at 37 °C for 1 h. Finally, cyclosporine was added to selectively kill T lymphocytes and generate only a cell line of B lymphocytes as detailed previously.^2^ In short, LCLs were grown in RPMI 1640 Medium, GlutaMAX Supplement (61870044; Thermo Fisher Scientific), supplemented, and fortified with 10% fetal bovine serum (FBS; Biochrom), L-glutamine (P04-80050; PAN Biotech), and antibiotics (penicillin/streptomycin, P06-07050; PAN Biotech). Cells were cultured at 37 °C in incubators supplied with 5% CO_2_.

### Immunofluorescence and immunoblotting

To localize and visualize proteins in cells, we used immunofluorescence (IF). For this purpose, coverslips were coated with poly-l-lysine hydrobromide 0.1% (P5899-5MG; Sigma-Aldrich) for 10 min prior to seeding the LCLs. After achieving 70% confluency, cells were fixed either by methanol or 3% paraformaldehyde (PFA). After permeabilization with 0.5% Triton X-100, cells were blocked in 5% FBS for 1 h. Notably, methanol fixed cells were not permeabilized, they were directly subjected for blocking after fixation. In the next step, cells were incubated overnight with primary antibodies, followed by incubation with secondary antibodies and DAPI for 30 min in the dark. Finally, cells were mounted on a glass slide with the help of Fluoromount-G Mounting Medium (00-4958-02; Thermo Fisher Scientific). For imaging, confocal laser scanning microscope (TCS SP8 gSTED; Leica Microsystems) was used.

For immunoblotting (IB), cells were collected and lysed using radioimmunoprecipitation assay (RIPA) buffer (50 mM Tris-HCl [pH 7.5], 0.1% Triton X-100, 150 mM NaCl, 0.5% Na-deoxycholate, 0.1% sodium dodecyl sulfate [SDS]) along with Protease Inhibitor Cocktail (PIC; P8340; Sigma-Aldrich) and 1 mM each of protease inhibitors DTT, benzamidine, and PMSF. The cells were subsequently homogenized by passing through the 0.4 × 19 mm syringe attached with needles (27G × 3/4″, Nr.20, BD Macrolane TM3) and incubation on ice for 15 min. After centrifugation, proteins were denatured at 95 °C in 5× SDS sample buffer. Resultant proteins were resolved by 4–12% SDS-PAGE (EC-890; National Diagnostics) and transferred to nitrocellulose membrane (PROTRANR, Germany). After blocking in 5% milk powder, membranes were incubated with primary antibodies overnight at 4 °C and respective secondary antibodies for 1 h at room temperature. Finally, proteins were visualized on X-ray films using an enhanced chemiluminescence (ECL) system. The detailed procedure of IF and IB was also reported previously.^2^

The following primary antibodies were used for IF and IB: rabbit polyclonal CK2β (Ab76025 for IF; Abcam), mouse monoclonal CK2β (Sc-12739 for IB; Santa Cruz Biotechnologies), mouse monoclonal β-catenin (05-665, active form dephosphorylated on Ser33, Ser37, and T41; Millipore),^28^^,^^29^ rabbit monoclonal β-catenin (ab32572, non-active form phosphorylated on Ser33, Ser37, and T41; Abcam),^28^^,^^29^ rabbit monoclonal DVL3 (ab76081; Abcam), mouse monoclonal α-tubulin (T8328; Sigma-Aldrich), rabbit polyclonal Lamin A/C (H-110, sc-20681; Santa Cruz Biotechnology), mouse monoclonal GAPDH peroxidase-conjugated (G8795 for IB; Sigma), mouse monoclonal anti-GFP (K3-184-2),^30^ and in-house manufactured mouse monoclonal glutathione S-transferase (GST).^31^ Secondary antibodies used for IF were Alexa Fluor 488 donkey anti-rabbit IgG (A21206; Invitrogen), Alexa Fluor 568 goat anti-mouse IgG (A11004; Invitrogen), and Alexa Fluor 568 donkey anti-rabbit IgG (A11004; Invitrogen). Secondary antibodies for IB were anti-mouse IgG peroxidase conjugate (A4416; Sigma-Aldrich) and anti-rabbit IgG peroxidase conjugate (A6154; Sigma-Aldrich).

### Fractionation assay

The cells were washed with PBS, trypsinized, and pelleted at 1,100 rpm at 4 °C. The cells’ pellet was dissolved in PBS containing 0.1% Nonidet P-40 (NP-40). One-third of the dissolved lysate was taken out and processed as whole-cell lysate. To separate out the cytoplasmic fraction, we centrifuged the dissolved cell lysate at 15,000 rpm for 10 s at 4 °C; half of the supernatant was taken out and boiled with SDS sample buffer. Next, the pellet was washed with 1 mL of PBS containing 0.1% NP-40 and centrifuged. The supernatant was discarded again, and the rest of the pellet was boiled with SDS sample buffer, which was taken as the nuclear fraction.

### Plasmids construction for protein purification and transient expression

The open reading frames (ORFs) of *CSNK2B* (GenBank: NM_001320.7) and *CSNK2A1* (GenBank: NM_177559.3) were cloned in pGEX4T1 (GE Healthcare) and pEGFP-C1. Identified variants of *CSNK2B* GenBank: NM_001320.7, c.94G>C, c.94G>A, and c.374G>C, were introduced in these plasmids by site-directed mutagenesis (Promega). Oligonucleotides sequences used to create variants of *CSNK2B* are shown in Table S2. We obtained GFP-CTNNB1 plasmid as a donation from Prof. Carien Niessen (Cologne Excellence Cluster on Cellular Stress Responses in Aging-Associated Diseases [CECAD], University of Cologne). This plasmid contains the ORF of *CTNNB1* (GenBank: NM_001904.4), which was excised and introduced into pGEX4T1 plasmid.

### Protein expression in eukaryotic cells

Eukaryotic expression plasmids (pEGFP-C1) of both wild-type and mutants *CSNK2B* and *CTNNB1* were transfected in HeLa cells using 1 mg/mL polyethylenimine (PEI; 23966; Polysciences) or Lipofectamine 2000 (11668019; Thermo Fisher Scientific). Cells were subjected to IF, IB, and/or pull-down assay after 24 h of transfection.

### GST fusion protein expression and purification

To obtain the bacterially expressed recombinant *Homo sapiens* CK2β, CK2α, and Catenin beta-1 proteins, also known as β-Catenin, prokaryotic expression plasmids (pGEX4T1) containing ORFs of the respective genes were cloned and expressed in *E. coli* Arctic Express cells. After induction with 0.3–0.5 mM isopropyl beta-D-1-thiogalactopyranoside (IPTG), cells were grown at 10 °C overnight. Bacterial cell pellets were resuspended in ice-cold STE buffer (10 mM Tris-HCl [pH 8.0], 150 mM NaCl, and 1 mM EDTA) mixed with PIC (P8340; Sigma-Aldrich), protease inhibitors (1 mM each of DTT, benzamidine, and PMSF), lysozyme (100 μg/mL), and 1% Sarcosyl. After sonication and centrifugation, the supernatant was mixed with 2% Triton X-100. The protein was purified by stimulating binding to Glutathione Sepharose 4B beads (GE17-0756-01; GE Healthcare) by overnight incubation in STE buffer mixed with PIC and 1 mM each of DTT, benzamidine, and PMSF.

All the GST-tagged recombinant proteins (Figure S1A) were subjected to cleave GST (Figure S1B) with the help of thrombin (T6634; Sigma-Aldrich).

### Pull-down assay and mass spectrometry analysis

To assay the possible impaired interaction between mutant CK2β and β-Catenin, we incubated Glutathione Sepharose 4B beads carrying GST-tagged CK2β (wild type and mutants) with precleared cell lysates of HeLa cells expressing GFP-tagged β-Catenin. To study impact of variant on global interaction of CK2β, we added Glutathione Sepharose 4B beads carrying GST-tagged CK2β (wild type and mutants) to the precleared HeLa cell lysates.

In all cases, overnight incubated beads were sedimented, washed five times with PBS containing 1× PIC and 1 mM each of DTT, benzamidine, and PMSF. The interaction was either analyzed by IB or samples were subjected to mass spectrometry (MS). MS data were analyzed on the basis of log_2_ LFQ (label-free quantification; signifies the relative amount of proteins in two or more biological replicates) intensities and number of peptides pulled down with purified GST-tagged CK2β wild type and mutant. Proteins obtained for negative control (GST-only) were excluded from the analyses. Pathway enrichment of resultant proteins were performed by FunRich (functional enrichment analysis tool, version 3.1.3).

### Microscale thermophoresis

To monitor the effect of variant on interaction of mutant CK2β with CK2α, we performed microscale thermophoresis (MST). Purified CK2α, containing unnatural amino acid *para*-Azidophenylalanine (*p*AzF) at Tyr239, was coupled with a fluorophore DBCO-Sulfo-Cy5 via strain-promoted azide - alkyne click chemistry reaction (SPAAC) click reaction.^32^ Serial dilution ranging from 0.1526 to 5,000 nM GST cleaved wild-type and mutant CK2β was mixed with 30 nM modified CK2α in final buffer containing 50 mM Tris-HCl (pH 8), 500 mM NaCl, 500 μM CaCl_2_, 0.05% Tween 20, and 0.5% (v/v) DMSO. Thermophoretic movement of fluorescently labeled CK2α was measured by monitoring the fluorescence distribution inside a capillary of Monolith NT.115 (NanoTemper, Munich, Germany). Fluorescence (red filter, light emitting diode [LED] power 50%) and thermophoresis (MST power 20%) were recorded at 25 °C. The dissociation constant (K_D_) values were determined from several independent experiments using NT Analysis v.2.1.3 software (NanoTemper Technologies, Germany).

### Kinase assays

To investigate the impact of variants on the kinase activity of CK2 holoenzyme, we conducted two different kinds of assay; their details are given below.

#### ADP-Glo assay

Phosphorylation of β-catenin by CK2 was analyzed using a luminescent ADP detection assay (Promega, Madison, WI, USA). Tetrameric CK2 holoenzyme was initially reconstituted by mixing 100 nM CK2α with 200 nM CK2β (wild type or one of both mutants, NP_001311.3; p.Asp32His and NP_001311.3; p.Asp32Asn) in 80 mM NaCl, 280 mM Tris-HCl (pH 8.0), 50 mM CaCl_2_, 7.5 mM MgCl_2_, and 1 mM ATP. After adding 6 μM β-catenin to a total volume of 5 μL, the reaction mixture was incubated for 25 min at 37 °C. Afterward, 5 μL of ADP-Glo reagent was added to terminate the kinase reaction. After 40 min of incubation at room temperature, 10 μL of kinase detection reagent was added to convert the generated ADP into ATP, which was measured in a luciferase/luciferin reaction. Luminescence was detected after 1-h incubation at room temperature with a Tecan Infinite M200 Pro (Tecan, Männedorf, Switzerland).

#### Capillary electrophoresis

CK2 activity was analyzed using a previously published capillary electrophoresis (CE)-based method.^33^ For kinase reaction, kinase buffer (50 mM Tris-HCl [pH 7.5], 100 mM NaCl, 10 mM MgCl_2_) containing 70 nM CK2α and 140 nM CK2β (wild type and both mutants NP_001311.3; p.Asp32His and NP_001311.3; p.Asp32Asn) was pre-incubated for 10 min at 37 °C. After adding pre-incubated assay buffer (25 mM Tris-HCl [pH 8.5], 150 mM NaCl, 5 mM MgCl_2_, 190 μM substrate peptide RRRDDDSDDD^34^ [GenicBio, Shanghai, China] and 60 μM ATP), the samples were incubated at 37 °C for 1, 2, 3, 4, and 5 min. The reaction was stopped by decreasing the temperature to 4°C and addition of 5 μL EDTA (0.5 M). Samples were analyzed by Beckman Coulter pa800 plus (Krefeld, Germany) CE system, with 2 M acetic acid (pH 2.0) as background electrolyte and a constant current of 30 μA. Peptides were detected at a wavelength of 195 nm.

### RNA extraction, quantitative real-time PCR, and transcriptome profiling

Total RNA extracted from LCLs using RNeasy Mini kit (74104; Qiagen) was converted into cDNA using SuperScript II reverse transcriptase (RT) enzyme (18064014; Invitrogen). We performed quantitative real-time PCR using an already described method.^35^ Oligos are enlisted in Table S2.

For whole-transcriptome profiling, we subjected 1 μg of total RNA for poly(A) selection. After mRNA fragmentation, adaptor ligation, and cDNA synthesis, libraries were sequenced on Illumina HiSeq4000 (Illumina) with a 2 × 75-bp read length. Obtained data were processed through the QuickNGS pipeline of CECAD.^36^ Reads were mapped to the *Homo sapiens* reference genome version GRCh37 using TopHat2 (version 2.0.10)^37^ and abundance estimation with Cufflinks (version 2.1.1).^38^ DESeq2 is used for differential gene expression analysis.^39^ The results were uploaded into the QuickNGS database for further analyses. Genes were filtered on the basis of involved pathways. Heatmaps were generated based on FPKMs (fragments per kilobase per million) values, using Heatmapper^40^ to visualize the DEGs (differentially expressed RefSeq genes). Pathway enrichment was performed using PANTHER (protein analysis through evolutionary relationships).

### Whole-phosphoproteome profiling

A total of 3 mg of protein lysates obtained from *CSNK2B* mutated individual (subject 1) LCLs, along with wild-type (three biological replicates of both) LCLs, was subjected to phosphopeptide enrichment (PPE). The isolation of phosphopeptides was carried out by a previously described method with some modifications.^41^ Further, the lysates were incubated with DTT and iodoacetamide and digested overnight using trypsin (1:50 w/w ratio). Then, the peptides were desalted and passed through a polysulfoethyl column (4.6-mm inner diameter [ID]×20-cm length, 5-μm particle size, 300-Å pore size; PolyLC, Switzerland) for cation exchange fractionation. The gradient being used in this experiment was solvent A (composition given below) (100%) and solvent B (0%) for 2 min, followed by addition of solvent B (0–20%) for 40 min, increasing the concentration of solvent B (20–100%) for 5 min; finally, 100% solvent B held for 5 min was processed. The composition of solvent A was 5 mM KH_2_PO_4_ 25% acetonitrile (ACN) (pH 2.7), and solvent B was 5 mM KH_2_PO_4_, 25% ACN, 350 mM KCl (pH 2.7). Based on the number of peptides, the fractions of 5 up to 10 were being collected.

PPE was performed using FeNTA-IMAC columns (Pierce). Cleaning of phosphopeptides was performed using ZipTips followed by their submission to nano-scale liquid chromatographic tandem mass spectrometry (nLC-MS/MS) analyses on LTQ Orbitrap machine. The fractionation of peptides was carried out via nLC on a 150-mm C18 column (75-μm ID; Dr. Maisch GmbH, Ammerbuch, Germany) using an EASY nLC-II system (Proxeon/Thermo Fisher Scientific). The separation of peptides was performed at 300 nL/min flow rate for 90 min (5%–7% ACN in 5 min, 7%–45% in 60 min, 45%–50% in 5 min, 50%–97% in 5 min; wash at 100%). Here the composition of buffer A was 0.1% formic acid dissolved in H_2_O, and buffer B contained 0.1% formic acid diluted in acetonitrile. The survey full-scan MS spectra (m/z 300–2,000) of intact peptides was carried out in the Orbitrap at a resolution of 30,000 using m/z 445.12003 as a lock mass.

The mass spectrometer was used for spectra in data-dependent automatic mode (automatic switch between MS and MS/MS acquisition). Further, dynamic exclusion was enabled for 1 min. The five most intense ions having a charge state z ≥ 2 were isolated and fragmented in the linear ion trap; this was carried out by collision-induced dissociation fragmentation. However, the peptides having unknown z values were not being fragmented.

### Bioinformatics analyses for phosphoproteome profiling

Andromeda, a peptide search engine, was used for searching the RAW files followed by searching against the UniProtKB database. The identification criteria of peptides were two miscleavages; minimum peptide size of seven amino acids; variable modifications were methionine oxidation, serine/threonine/tyrosine phosphorylation, and protein N-terminal acetylation; and fixed modifications were alkylation of cysteine. Furthermore, up to three post-translational variable modifications were selected.

For detecting parent ions, first search mass accuracy was 20 ppm, with the second being 4.5 ppm. Fragment ion match was 0.5 Da. Phosphorylation sites with a localization probability >0.75 (class I phosphorylation sites) and a delta score >40 were preferably selected and used for further analyses. Other analyses were performed as described elsewhere.^42^

To generate the position weighted matrices, we used iceLogo. The normal amino acid distribution of the respective species was considered as background.^43^ Furthermore, netphorest 2.0 was used in order to predict potential kinase substrates.^44^ NCBI HomoloGene groups (CPhos program) were used for identifying the conservation.^45^^,^^46^ Gene Ontology enrichment was carried out by GORilla using the mouse homologous gene symbols, which were taken from CPhos/HomoloGene.^47^ The p value threshold was 10^−3^, and false discovery rate (FDR)-corrected q-values were reported. Sequences were predicted by using different calculation methods as described elsewhere;^48^ predict protein suite, IUPRED, and FoldIndex were used in this study. The protein region denominated “intrinsically disordered” in all prediction methods was chosen. For the correlational analyses of phosphorylation site abundance, Perseus was used.^49^

## Results

### Description of subjects

**Figure 1:**
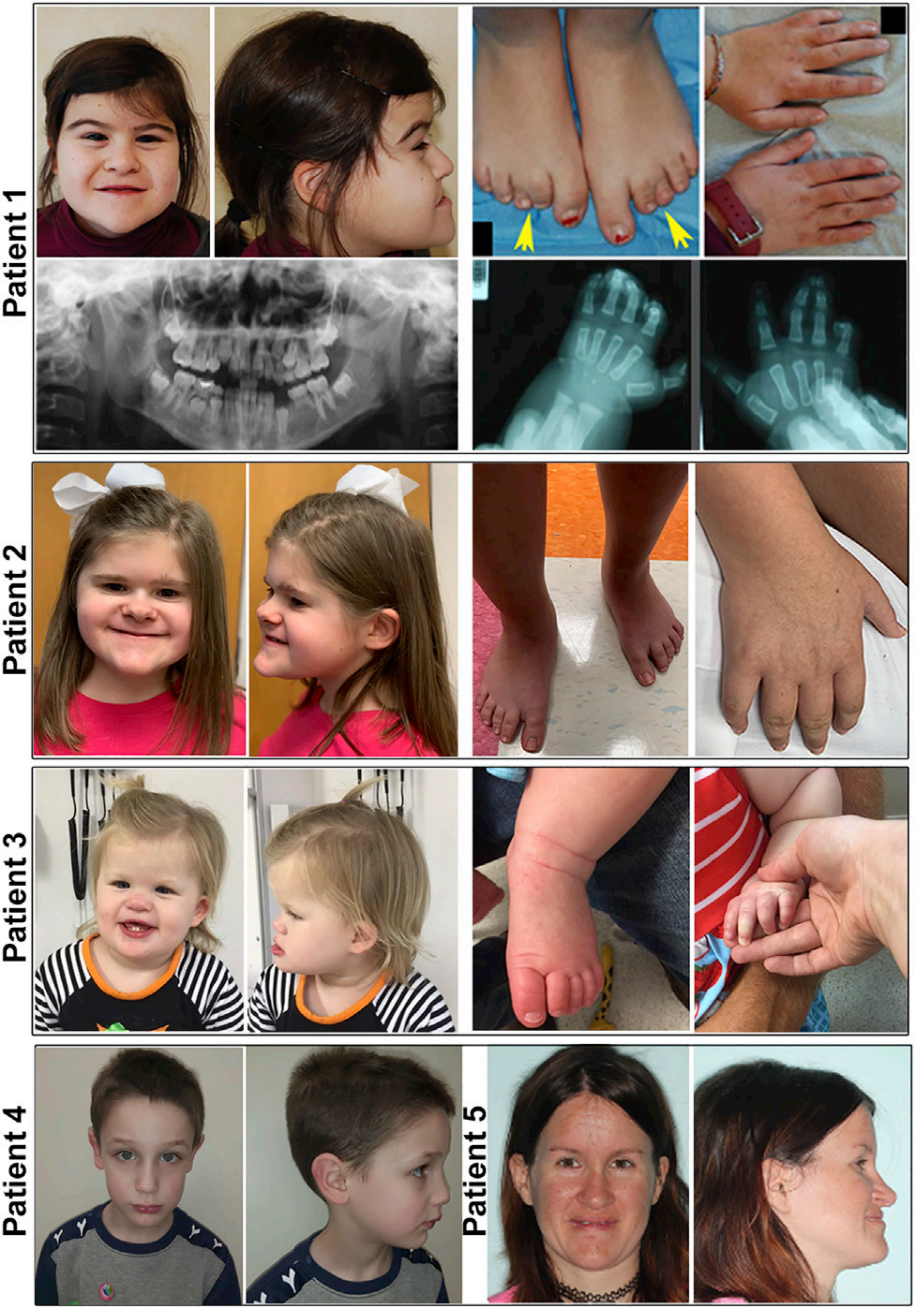
Clinical presentation of patients with *CSNK2B* variants Front and side views of subjects 1–3 diagnosed with IDCS show strikingly similar facial gestalt, whereas subjects 4 and 5 diagnosed with POBINDS show vivid difference of facial gestalt, and no obvious digital or limb anomaly was recorded as in IDCS. Digital anomalies of IDCS patients are shown in two panels on right side. For subject 1, arrows show brachydactyly 2–5 and syndactyly 2–3 of the digits of the feet. Notably, preoperative radiographs of the hands and teeth are shown for subject 1.

We report on five patients with pathogenic *CSNK2B* variants. Three of them are manifesting a novel IDCS, while the remaining two affected individuals, subjects 4 and 5, are sharing phenotypic similarities with POBINDS (MIM: 618732) (Figure 1; Table 1).

**Table 1:** Clinical findings of patients with *CSNK2B* variants

Individuals	Subject 1	Subject 2	Subject 3	Subject 4	Subject 5
*CSNK2B* variant (GenBank: NM_001320.7)	c.94G>C (p.Asp32His)	c.94G>A (p.Asp32Asn)	c.94G>A (p.Asp32Asn)	c.374C>G (p.Ser125∗)	c.367+5delG (p.Leu124Aspfs∗26 and p.Leu98Alafs∗11)^a^
Inheritance	*de novo*	*de novo*	unknown	*de novo*	unknown
Sex	female	female	female	male	female
Age (years)	19	10	2	7	30 (deceased)
Ancestry	Caucasian	Caucasian	Caucasian	Israeli	Caucasian
Parenteral consanguinity	–	–	–	–	–
Measurements (at birth)					
Gestational age (weeks)	41	39	39	40	N/A
Height (SD)	−1.9	−0.8	−2.8	N/A	−1.33
Weight (SD)	−1.3	−1.1	−0.8	N/A	0.21
OFC (SD)	−0.9	N/A	−1.2	N/A	N/A
Measurements (at age)	12 years	9 years	2 years	6 years	30 years
Height (SD)	−3.3	+0.11	−0.26	−0.32	−0.8
Weight (SD)	−1.9	+1.53	+0.21	−0.41	−2.03
OFC (SD)	−1.7	+0.9	−0.24	+0.36	N/A
Neurological features					
Global developmental delay	+	+	+	+	+
Intellectual disability	+ (moderate)	+	Developmental delay	+	+
Speech impairment	+ (moderate)	+	+	+	N/A
Epileptic seizures	+ (tonic-clonic)	EEG abnormalities	–	+	+ (tonic-clonic, myoclonic, clonic, absence seizures)
Facial features					
Deep-set eyes	+	+	+	–	eye movement issues, hyperopia, myopia, lazy eye
Nasal bridge	broad	depressed	broad, depressed	prominent	prominent
Hypoplastic alae nasi	+	+	+	+	+
Mouth	thin lips	thin lips	arched upper lip	–	–
Prognathism	+	+	+	–	+ (class III underbite)
Pointed chin	+	+	+	–	+
Ears	small, prominent antitragus	symmetric protruding	asymmetric: left cupped, right overfolded	protruding	–
Hands and feet					
Fingers	brachydactyly cutaneous syndactyly of fingers 3/4/5 (required surgery) including osseous syndactyly of distal phalanges 3/4	tapering of fingers, most prominent on fifth fingers	brachydactyly contractures of the left hand first, fourth, and fifth fingers (required surgery on first and fourth); initial reports of mild right-sided first and second digit involvement	–	–
Toes and feet	brachydactyly 2–5 syndactyly 2–3	clinodactyly of toes 3/4/5 pronation of feet	small feet mild inversion and pronotion of feet	–	flat feet
Ectodermal anomalies					
Hair	thin	thin	sparse temporal, anterior thin, posterior coarse	hypopigmentation, partial	–
Teeth	hypodontia	small teeth	delayed eruption	–	
Cardiac defect	Ebstein’s anomaly and atrial septal defect	WPW	fenestrated atrial septal defect	mild cardiac defect	–
Others		IgA nephropathy; nocturnal enuresis		emotional regulation disorder, low attention span	hypotonia, low tone, memory impairment, anxiety, depression, obsessive compulsive disorder, proprioception issues

+, present; −, not present/no abnormality; N/A, not available; OFC, occipital-frontal circumference; SD, standard deviation; WPW, Wolff-Parkinson-White.

a Variants at protein levels resulted from minigene splicing assay.

To investigate the facial gestalt differences of our IDCS affected members (subjects 1–3) with those of POBINDS, we employed GestaltMatcher. Additionally, we included two previously reported patients; one (subject 6) manifesting POBINDS^23^ and the other subject 7 (his original ID in the published article is patient 25) carrying the CK2β: NP_001311.3; p.Asp32Asn variant.^8^ The pairwise ranks of seven photos showed remarkable facial similarities among subjects 1, 2, 3, and 7, and hence they are grouped together in one cluster. Notably, subjects manifesting POBINDS showed considerable differences and were placed far away from subjects 1–3 and 7 (Figure S2). Interestingly, subjects 1 and 2 appeared closer to each other as compared with subjects 3 and 7. Subject 1 was at the first rank of subject 2, and subject 3 was at the 17th rank of subject 2, indicating a high degree of similarity in the phenotype (Figure S2). In short, the results suggested a novel phenotype seen in subjects 1–3 and 7 that is remarkably different from patients manifesting POBINDS. Detailed clinical features of these affected members are described below.

Subject 1 is a 19-year-old Italian woman born from healthy and unrelated parents (Figure 1; Table 1). She had short stature of −3.3 standard deviation (SD) (HP: 0004322); however, microcephaly (HP: 0005484) was not observed. She exhibited global developmental delay (HP: 0001263), moderate intellectual disability (HP: 0002342), dysarthria (HP: 0001260), and epileptic seizures (HP: 0001250). Digital findings included hypoplasia of fingers (HP: 0006265) and toes (HP: 0010173), cutaneous syndactyly of fingers 3–5 bilaterally (HP: 0010554); X-ray radiographs demonstrated bilateral osseous syndactyly of the distal phalanges of the third and fourth fingers (HP: 0010492), clinodactyly (HP: 0001863), bilateral syndactyly of second and third toes (HP: 0001770), and broad thumb (HP: 0011304). Notable facial dysmorphic features (HP: 0001999) included deep-set eyes (HP: 0000490), broad nasal bridge (HP: 0000431), hypoplastic alae nasi (HP: 0000430), thin upper lip (HP: 0000219), prognathism (HP: 0000303) with pointed chin (HP: 0000307), and small ears (HP: 0008551) with prominent antitragus (HP: 0008593). Further, ectodermal anomalies were observed such as hypodontia (HP: 0000668) depicted by dental radiographs (Figure 1) and thin hair (HP: 0008070). Ebstein’s anomaly (HP: 0010316) and atrial septal defect (HP: 0001631) were also identified in this patient (Table 1).

Subject 2 is a 9-year-old Caucasian girl with developmental delay, intellectual disability (HP: 0001249), and speech impairment (Figure 1; Table 1). Electroencephalogram (EEG) of this patient demonstrated excessively fast frequencies and interictal discharges (posterior predominant), indicative of generalized or mixed epilepsy (HP: 0001250). Among digital anomalies, syndactyly (HP: 0001159) was absent, yet other features, including bilateral tapering of fingers (HP: 0001182) (most prominent on fifth digits), bilateral clinodactyly (HP: 0001863) of third to fifth toes, and protonation of both feet were apparent. In addition, a 1- to 2-cm poorly circumscribed firm mass on plantar aspect of left heel was also notable. She presented facial dysmorphism (HP: 0001999), including deep-set eyes (HP: 0000490), hypoplastic alae nasi (HP: 0000430), thin upper lip (HP: 0000219), prognathism (HP: 0000303) with pointed chin (HP: 0000307), and asymmetric ears (HP: 0010722)—the right ear was more prominent than left.

Subject 3 is a 2-year-old Caucasian girl (Figure 1; Table 1). Initial physical examination at the age of 10 months revealed a large fontanelle (HP: 0000239) (4 cm), epicanthal folds (HP: 0000286), telecanthus (HP: 0000506), depressed nasal bridge (HP: 0005280), broad nasal tip (HP: 0000455), small cupped ears (HP: 0000378), which are low set, arched upper lip, widely spaced nipples (HP: 0006610), brachydactyly (HP: 0001156), and contracture of fourth and fifth fingers of left hand. At the age of 2 years, she exhibited salient facial features similar to subjects 1 and 2, such as deep-set eyes (HP: 0000490), hypoplastic nasal alae (HP: 0000430), and prognathism (HP: 0000303). She presented with developmental delay. Growth parameters were within normal ranges. No seizures were observed. The subject presented digital abnormalities, such as contractures of the fourth and fifth fingers, which required surgery (a trigger finger release). Dental examination showed delayed eruption of teeth (HP: 0000684), i.e., presence of only two lower teeth with two more emerging on the bottom and possibly two on the top. A fenestrated atrial septal defect (HP: 0001631) was present.

Clinical features of subjects 4, 5, and 7 are given in the supplemental information.

### Identification of *CSNK2B* variants

**Figure 2:**
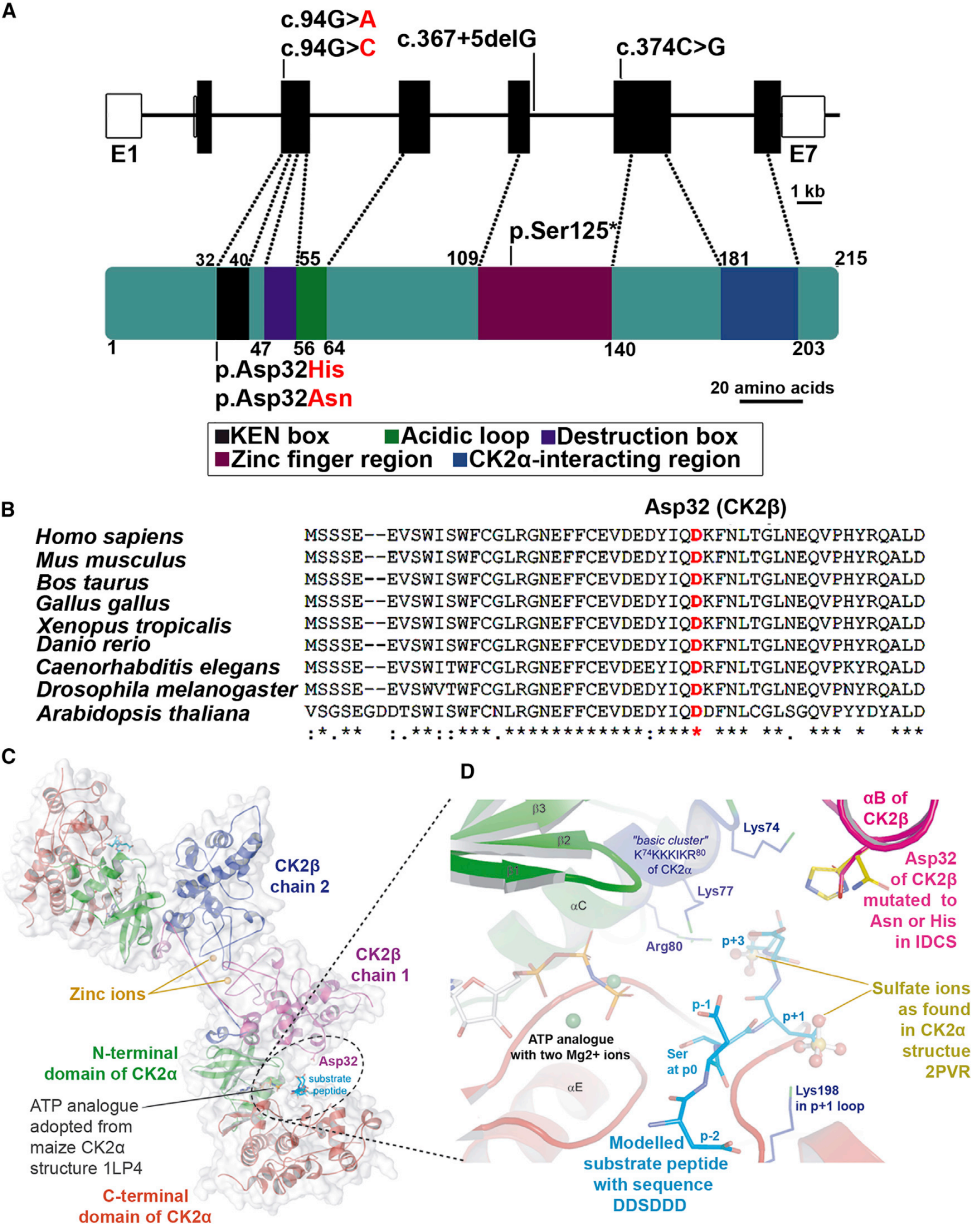
*CSNK2B* variants identified in IDCS patients (A) Upper panel: genomic structure of human *CSNK2B*. The seven exons of *CSNK2B* are displayed by boxes, which are drawn to scale (1 kb = 1 cm). The filled boxes depict the open reading frame, and the open boxes show the UTRs. The introns are drawn as connecting lines of arbitrary length. Vertical lines on filled boxes indicate the positions of the variants. Lower panel: 215-amino-acid-long CK2β protein composed of five domains along with identified variants are shown. The protein structure is constructed according to the indicated scale bar. Figure is recreated from Bibby and Litchfield (2005).^12^ (B) Cross-species alignment showing conservation of Asp32 of CK2β. Note that Asp32 is conserved in all species. Asterisk (∗) is used for the conserved residues, colon (:) for conservative changes, and dot (.) for semiconservative changes. (C) Structural overview of the CK2α2β2 holoenzyme along with a modeled substrate peptide and the ATP analogue AMPPNP. Dotted circle shows the wild-type amino acid Asp32 and substrate peptide in its close proximity. (D) Zoomed picture focuses on the critical neighborhood around Asp32 of CK2β illustrating the IDCS-associated variants Asp32Asn and Asp32His. Two sulfate ions visible in the human CK2α structure PDB: 2PVR^26^ were drawn in ball-and-sticks representation; as outlined in the Material and methods section, these sulfate ions served as an orientation to model the p+1 and p+3 side chains of the substrate peptide DDSDDD (blue carbon atoms) into the active site.

For identifying the disease-causing variant(s) in family 1, the following strategy was adopted; due to phenotypic overlap of patient 1 with Filippi syndrome, we first excluded pathogenic variants in *CKAP2L* by Sanger sequencing.^2^ In a next step, we performed trio exome sequencing of subject 1 and both parents. Data revealed *de novo* variants in *CSNK2B* (GenBank: NM_001320.7:c.94G>C (p.Asp32His)) and *MTM1* (MIM: 300415, GenBank: NM_000252.2:c.1186T>G (p.Phe396Val)) (Figure 2A). Because *de novo* variants in *CSNK2B* have been reported to cause intellectual disability and epilepsy,^50^, ^51^, ^52^ while *MTM1* has been linked to X-linked myotubular myopathy,^53^ we considered *CSNK2B* more interesting to investigate its contribution to the disease. Causative structural variants were excluded by molecular karyotyping using the CytoScan HD array from Thermo Fisher Scientific. In addition, subjects 2–5, carrying variants in *CSNK2B*, were recruited through GeneMatcher and GenomeConnect.^20^^,^^21^ In all of them, exome sequencing revealed variants in *CSNK2B*, which were confirmed *de novo* in all except subjects 3 and 5, because parental samples were not provided for testing, the same missense variant (GenBank: NM_001320.7:c.94G>A (p.Asp32Asn)) in subjects 2 and 3, a nonsense variant (GenBank: NM_001320.7:c.374C>G (p.Ser125∗)) in subject 4, and a splice variant (GenBank: NM_001320.7:c.367+5delG) in subject 5 (Figure 2A; Table 1).

Both *CSNK2B* missense variants are predicted to be pathogenic by *in silico* tools (Table S1). All four newly identified *CSNK2B* variants are absent in gnomAD, EVS, Iranome, the Greater Middle Eastern (GME) Variome, and our Cologne Center for Genomics (CCG) in-house dataset comprising >3,360 exomes. Two of these variants, c.94G>A and c.367+5delG, are reported in dbSNP (rs1554169984 and rs1583610622, respectively) because of entries in ClinVar (Table S1). To be mentioned here, c.94G>A has been recently reported to cause developmental delay and generalized epilepsy;^8^ however, the study was not only deficient of a detailed clinical presentation of individuals carrying this variant but also lacked any functional analysis and interpretation of the identified variant. Hence we took the opportunity and explored the functional consequences of the variants identified in the patients manifesting the novel syndrome.

Multiple sequence alignments of CK2β orthologs showed that the residue Asp32, which is mutated in subjects 1–3, is highly conserved among the vertebrates (Figure 2B). Furthermore, from analyses performed on X-ray structure, we concluded that within the CK2α_2_β_2_ holoenzyme structure, Asp32 of CK2β is not directly involved in the CK2α/CK2β interface (Figure 2C); however, it resides in the vicinity of the active site region of CK2α (Figure 2D).

### *CSNK2B*: NM_001320.7;c.367+5delG impairs splicing

To observe the consequences of *CSNK2B*: NM_001320.7;c.367+5delG on transcript splicing, we constructed minigene splicing assay. The RT-PCR performed on the cDNA produced a transcript of 320 bp in both wild type and mutant. However, two aberrant transcripts of approximately 465 and 244 bp were seen only in the mutant. Sanger sequencing of the transcript of 320 bp showed normal splicing (Figure S3A). Analysis of the abnormally sized transcript of 465 bp revealed that the *CSNK2B*: NM_001320.7;c.367+5delG variant eliminated the actual splice donor and acceptor sites, resulting in the retention of intron 5 (Figure S3B, left panel) and thus causing a shift in the reading frame and introducing an early termination codon (NP_001311.3; p.(Leu124Aspfs∗26)). Analysis of a 244-bp transcript revealed skipping of 76 bp (*CSNK2B*: NM_001320.7;c.292_367del;p.[Leu98Alafs∗11]), which corresponds to complete exon 5 (Figure S3B, right panel). This also resulted in the frameshift leading to introduction of the premature stop codon (NP_001311.3; p.Leu98Alafs∗11).

### Variants impair the localization and amount of CK2β

**Figure 3:**
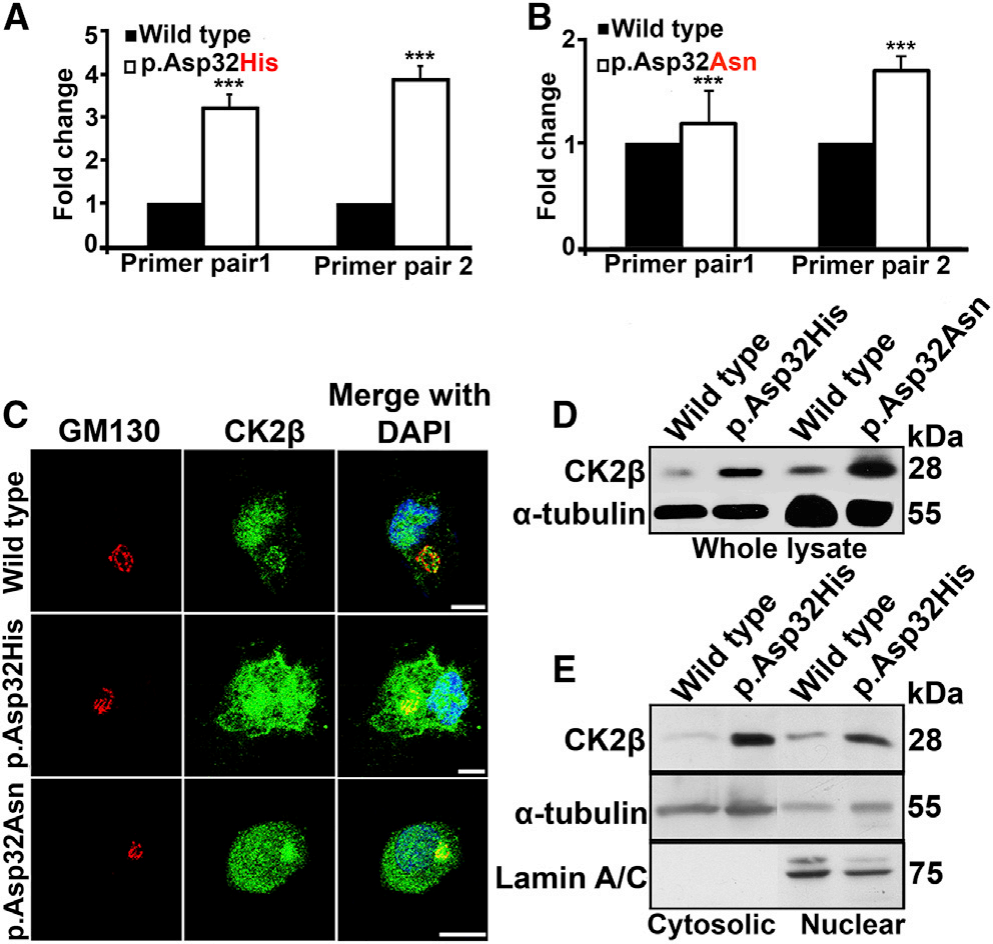
Quantification of *CSNK2B* transcript and encoded protein product along with cellular localization in LCLs (A) Quantitative real-time PCR data showing 3-fold increased expression of *CSNK2B* mRNA in patient (GenBank: NM_001320.7:c.94G>C (p.Asp32His)) as compared with wild type. *CSNK2B* mRNA amounts were quantified relative to control sample. ∗∗∗p = 0.0001 calculated by Student’s t test. n = 3; error bars represent standard deviation (SD). (B) Graph shows 2-fold increased expression of *CSNK2B* mRNA of subject (GenBank: NM_001320.7:c.94G>A (p.Asp32Asn)) resulting from amplifying a second set of primers as compared with wild type. ∗∗∗p = 0.0001 calculated by Student’s t test. n = 3; error bars represent SD. (C) Confocal microscopy images showing increased amount of CK2β (green) in nuclei and in cytoplasm of subject-derived (NP_001311.3; p.Asp32His and NP_001311.3; p.Asp32Asn) LCLs as compared with wild type. GM130 (red) serves as marker of Golgi apparatus, and DAPI (blue) indicates staining of nucleus. Scale bar: 10 μm. (D) Immunoblotting shows an increased amount of CK2β in whole-cell lysates obtained from mutant (NP_001311.3; p.Asp32His and NP_001311.3; p.Asp32Asn) LCLs versus wild type. α-Tubulin is used as loading controls. (E) Immunoblots show an increased amount of CK2β in cytosolic and nuclear fraction in NP_001311.3; p.Asp32His mutant versus wild type. Loading controls are α-tubulin and lamin A/C, for cytosol and nucleus, respectively.

As a first step to investigate the consequences of the identified variants, we performed a quantification of the *CSNK2B* transcripts in total RNA of subjects 1 and 2, which showed a significant upregulation of *CSNK2B* as compared with the control RNA obtained from LCLs (Figures 3A and 3B). We further analyzed cellular expression and sub-cellular localization of CK2β in control cells and patient-derived (subject 1: NP_001311.3; p.Asp32His and subject 2: NP_001311.3; p.Asp32Asn) LCLs. In wild-type LCLs, CK2β protein was observed to localize inside the nucleus, as well as at the Golgi apparatus, whereas both mutant LCLs showed excess of CK2β dispersed in the nucleus, as well as in the cytoplasm (Figure 3C). Furthermore, investigation of the abundance of CK2β by IB also showed an increased amount of CK2β proteins in the whole-cell lysate of LCLs derived from both subjects as compared with control (Figure 3D). To further quantify the CK2β in mutant NP_001311.3; p.Asp32His cellular fractions, we performed a fractionation assay coupled with IB. Our results showed an increased amount of CK2β in both cytosolic and nuclear fraction, as compared with the wild type (Figure 3E).

### Transiently expressed mutant CK2β mimics the results obtained with LCLs

To gain insights into the behavior of overexpressed wild-type and mutant CK2β proteins, GFP-tagged CK2β wild type and three mutants (NP_001311.3; p.Asp32His, NP_001311.3; p.Asp32Asn, and NP_001311.3; p.Ser125∗) were transiently expressed in HeLa cells. Similarly to endogenous CK2β observed in control LCLs, transiently expressed wild-type CK2β was found in the nucleus, as well as at the Golgi apparatus (Figure S4A). In contrast, two of the mutant proteins, NP_001311.3; p.Asp32His and NP_001311.3; p.Asp32Asn, were found in the cytosol and nuclei in the form of aggregates as tracked by GFP fluorescence (Figure S4A). Compared with the wild type, the mutant protein NP_001311.3; p.Ser125∗ was absent in cytosol, presumably because of the nonsense-mediated decay of the mutant mRNA. However, a drastically reduced nuclear expression was observed as compared with the wild type (Figure S4B). Notably, cells with ectopic expression of CK2β: NP_001311.3; p.Ser125∗ showed a distorted Golgi apparatus compared with the wild type and the remaining two mutants. These data were further corroborated by immunoblots where increased amounts of only two mutant proteins, NP_001311.3; p.Asp32His and NP_001311.3; p.Asp32Asn and a reduced amount of CK2β: NP_001311.3; p.Ser125∗ were noted compared with wild type (Figure S4C). Analysis of the band intensities indicates a 1.4- and 1.7-fold increase in case of NP_001311.3; p.Asp32His and NP_001311.3; p.Asp32Asn mutants, respectively, and a 0.2-fold decrease in case of the CK2β: NP_001311.3; p.Ser125∗ (Figure S4D).

### Interaction with CK2α is not impaired by missense variant

**Figure 4:**
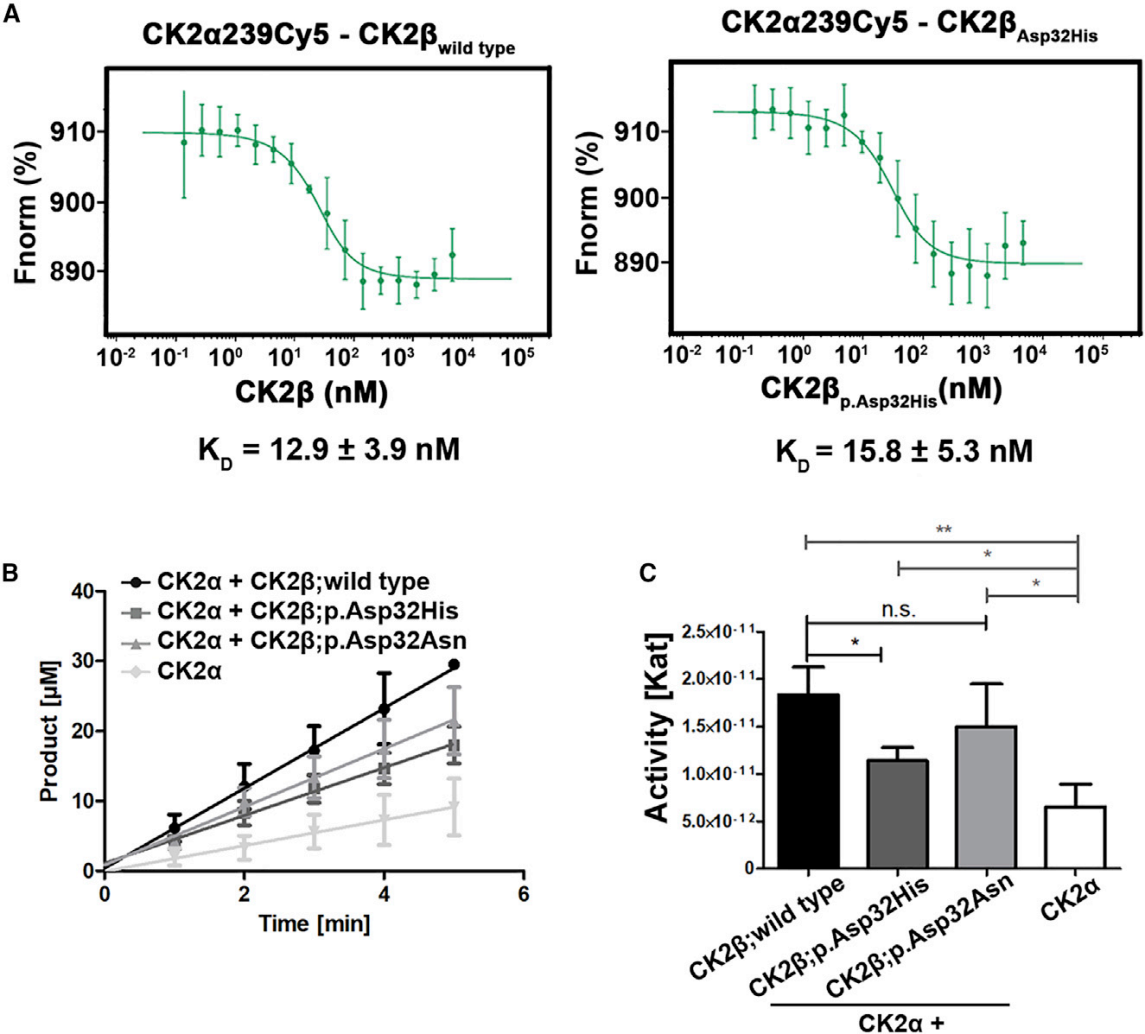
Interaction of CK2 subunits and kinase activity of mutated beta subunits (A) Graph shows interaction of CK2 subunits analyzed by microscale thermophoresis (MST). Graph in the left panel shows K_D_ value (12.9 ± 3.9 nM) in wild-type CK2β bound to CK2α239_Cy5_. n = 3. Graph in the right panel shows K_D_ values (15.8 ± 5.3 nM) in mutant CK2β (NP_001311.3; p.Asp32His) bound to CK2α239_Cy5_. n = 5. (B) Graph shows the amount of phosphorylated substrate peptide in the given time from capillary electrophoresis assay. Note that CK2 containing CK2β: NP_001311.3; p.Asp32His shows decreased amount of phosphorylated peptide as compared with wild type, whereas CK2 containing NP_001311.3; p.Asp32Asn shows no significant differences. The color and shape key denoting all the samples given in the figure. n = 3; error bars represent SD. (C) Kinase activity of CK2 wild type and mutants based on capillary electrophoresis assay. Bar graph shows kinase activity of CK2 containing wild-type and mutant beta subunits (NP_001311.3; p.Asp32His and NP_001311.3; p.Asp32Asn). Note that the activity of CK2 containing NP_001311.3; p.Asp32His is reduced as compared with wild type, but CK2 containing NP_001311.3; p.Asp32Asn variant does not show any significant difference. ∗p ≤ 0.05, ∗∗p ≤ 0.01, ^ns^p ˃ 0.05 (Student’s t test). n = 3; error bars represent SD. ns, non-significant.

To investigate the effect of the CK2β variant NP_001311.3; p.Asp32His on the binding efficiency to CK2α, we performed MST. The results showed hardly any difference in the binding affinity of CK2α with mutant (NP_001311.3; p.Asp32His) CK2β. Binding affinity of wild-type CK2β with CK2α was K_D_ = 12.9 ± 3.9 nM, and that with mutant (NP_001311.3; p.Asp32His) CK2β was K_D_ = 15.8 ± 5.3 nM (Figure 4A). Hence we conclude that the variant (NP_001311.3; p.Asp32His) of CK2β does not have any influence on the interaction potential with CK2α.

### CK2 holoenzyme with NP_001311.3; p.Asp32His beta subunit showed lower kinase activity

Because the interaction between mutant CK2β and CK2α seemed not to be impaired, we hypothesized that the variants may affect the kinase activity of the holoenzyme. Furthermore, close proximity of Asp32 to the active site region of CK2α (Figure 2D) also prompted us to perform kinase assay. For this purpose, we performed a CE-based assay using the CK2 target peptide RRRDDDSDDD as a substrate.^34^ We monitored the amount of phosphorylated substrate at several time points, and data showed significant reduction of the kinase activity only for the CK2 holoenzyme containing the NP_001311.3; p.Asp32His CK2β subunit when compared with the wild-type CK2 holoenzyme (Figure 4B). The slopes of the corresponding curves were also used to calculate the kinase activities, in terms of Kat, of the different variants (Figure 4C). As expected, we observed a significant decrease in the kinase activity of the tetrameric CK2 holoenzyme constituted with mutant (NP_001311.3; p.Asp32His) CK2β as compared with the holoenzyme containing wild-type subunits only. Intriguingly, kinase activity of this particular mutant CK2 holoenzyme was comparable with the activity observed for CK2α alone, thus showing a drastic influence on the phosphorylation activity (Figure 4C). Surprisingly, we did not observe a significant difference in the kinase activity of the CK2 holoenzyme containing the other mutant (NP_001311.3; p.Asp32Asn) CK2β (Figure 4C).

### Significant effects of variant NP_001311.3; p.Asp32His on protein-protein interactome

After analyzing the kinase activity of the mutated CK2, we extended our analyses to explore the consequences of CK2β: NP_001311.3; p.Asp32His on the global interaction pattern. To accomplish this aim, we performed pull-down assays; MS analysis of the precipitate obtained after a pull-down of wild-type GST-CK2β in whole HeLa lysate identified 194 proteins (Figures S5A–S5C). Among them, 124 are novel protein partners, whereas 69 were already reported as CK2 substrates (Table S3).^54^^,^^55^ The mutant NP_001311.3; p.Asp32His showed an impaired interaction for 38 proteins (Figure S5C; Table S3). Among these 38 proteins, 25 are already reported partners of CK2 (Table S3). Pathway enrichment of the 38 proteins showing impaired interaction with mutant CK2β revealed their involvement in the degradation of β-catenin, Wnt signaling, apoptosis, ataxia-telangiectasia mutated (ATM), mammalian target of rapamycin (mTOR), and IL-3 signaling (Figure S5D). Notably, degradation of β-catenin (DVL1 and DVL3) was noted as one of the most significantly enriched pathways (p > 0.05) (Figure S5D); therefore, we followed it for comprehensive investigations. Interestingly, DVL1 and DVL3 have also been reported to be functionally linked with CK2^15^; therefore, we focused on DVL3 and β-catenin for further analyses.

### Missense variant NP_001311.3; p.Asp32His compromises the interaction with β-catenin and DVL3

**Figure 5:**
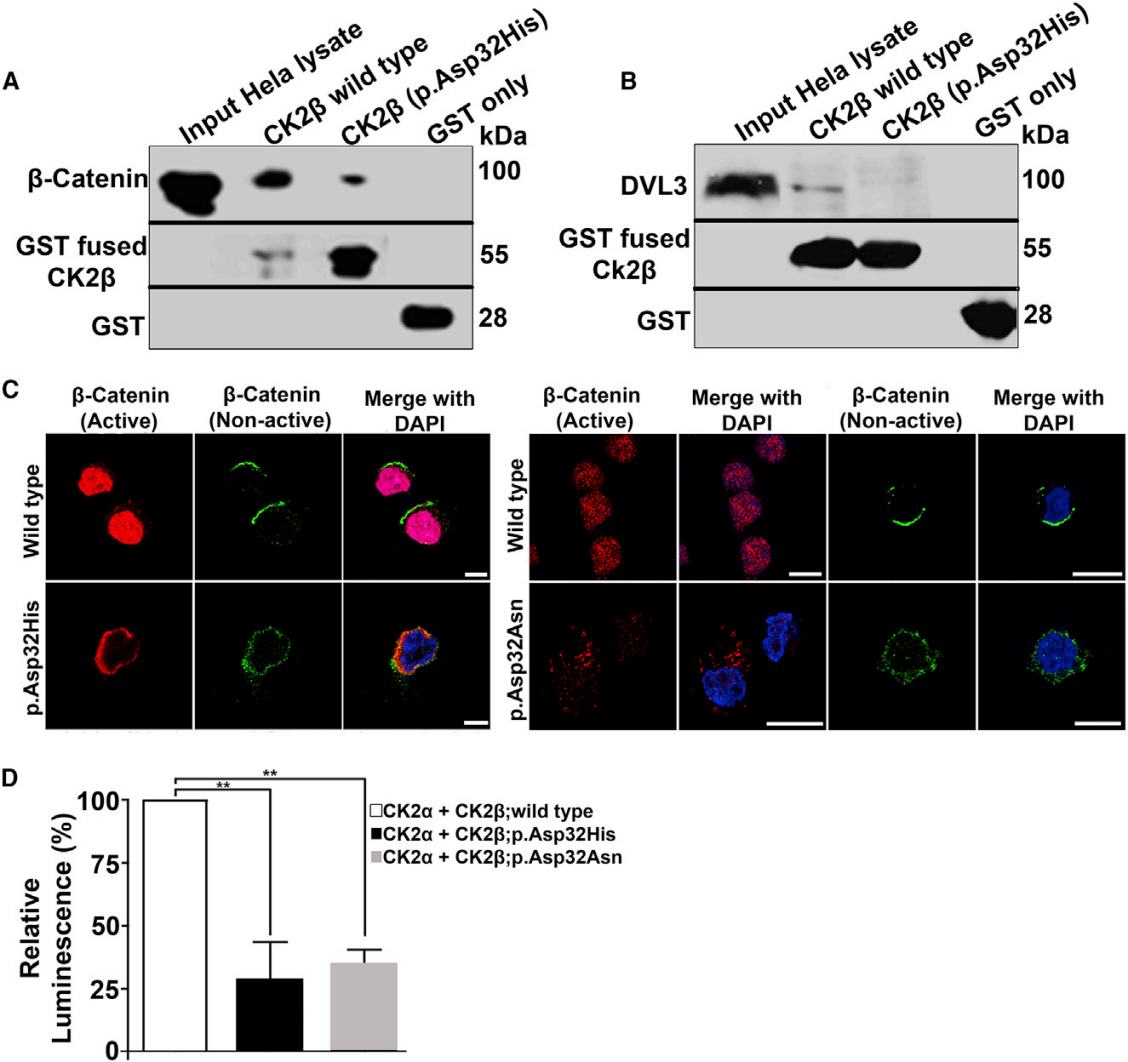
Impacts of variants on the dynamics of β-catenin and DVL3 (A) Pull-down assay from HeLa total protein extracts indicates reduced interaction of endogenous β-catenin with mutant (NP_001311.3; p.Asp32His) GST-tagged CK2β as compared with wild type. GST serves as negative control. Bands of approximately 85 kDa of β-catenin were observed on western blot after probing with rabbit monoclonal β-catenin antibody. GST-fused proteins were visualized by probing the membrane with in-house-generated mouse monoclonal GST antibody. (B) Pull-down assay shows reduced interaction of DVL3 with GST-fused CK2β mutant (NP_001311.3; p.Asp32His) as compared with wild-type. GST was used as negative control. Bands of approximately 78 kDa of DVL3 were observed by rabbit monoclonal DVL3 antibody. (C) Immunofluorescence shows decreased amount of active β-catenin (red) in nuclei of CK2β: NP_001311.3; p.Asp32His (left panel) and CK2β: NP_001311.3; p.Asp32Asn (right panel) LCLs as compared with the wild type. Localization pattern of non-active β-catenin (green) remains the same in wild-type and both mutant LCLs. DAPI (blue) indicates staining of nucleus. Scale bar, 5 μm (left panel); 10 μm (right panel). (D) Graph showing reduced kinase activity of CK2 carrying mutants (NP_001311.3; p.Asp32His and NP_001311.3; p.Asp32Asn) of CK2β as compared with wild type measured by ADP-Glo assay. Note that β-catenin was used as substrate. Error bars represent SD; n = 3. ∗∗p ≤ 0.01 (Student’s t test).

To assay the effects of the variants on the interaction of CK2β with DVL3 and β-catenin, GST-tagged CK2β wild-type and NP_001311.3; p.Asp32His mutant (Figure S1A) were pulled down with HeLa cell lysates. IB showed that the interaction of β-catenin with mutated GST-tagged CK2β was reduced as compared with wild type (Figure 5A). We also obtained similar results using another strategy where GST-tagged CK2β wild type and mutant were pulled down with HeLa cell lysates expressing GFP-tagged β-catenin (Figure S5E). Similarly, on analyzing the interaction of DVL3 with CK2β by pulling down wild-type and mutant (NP_001311.3; p.Asp32His) GST-tagged CK2β with protein lysate of HeLa cells, we noted considerably reduced interaction of DVL3 with mutant (NP_001311.3; p.Asp32His) CK2β compared with wild type (Figure 5B).

### β-Catenin is mislocalized in LCLs derived from subjects with missense variants

Impaired interaction of mutant CK2β with β-catenin and DVL3 prompted us to analyze the cellular expression of these proteins in subject-derived LCLs. IF analysis combined with confocal microscopy revealed the presence of an active/nuclear form of β-catenin inside the nuclei of wild-type LCLs, whereas it was absent in the nuclei of LCLs derived from both variant carriers (NP_001311.3; p.Asp32His and NP_001311.3; p.Asp32Asn); rather, it was found in the cytoplasm or near the periphery of the nucleus (Figure 5C). However, none of the mutant cells showed an alteration of the localization pattern of the cytoplasmic β-catenin (Figure 5C).

### Both missense variants reduce the phosphorylation capacity of β-catenin

Our data demonstrated the compromised interaction of mutant CK2β with β-catenin; therefore, we hypothesized that the impaired interaction may influence the kinase activity of the mutated holoenzyme and thus reduce the phosphorylation capacity of β-catenin. To this end, we performed the ADP-Glo assay by keeping β-catenin as a substrate. Data showed that the tetrameric holoenzyme containing mutant versions of CK2β had a reduced phosphorylation capacity of β-catenin by more than a half as compared with the wild-type tetrameric holoenzyme. Precisely, the data showed 72% and 65% reduction in the phosphorylation rate of β-catenin by NP_001311.3; p.Asp32His and NP_001311.3; p.Asp32Asn, respectively (Figure 5D).

### Whole-transcriptome profiling of mutant LCLs revealed drastic effects on differential expression of Wnt target genes

**Figure 6:**
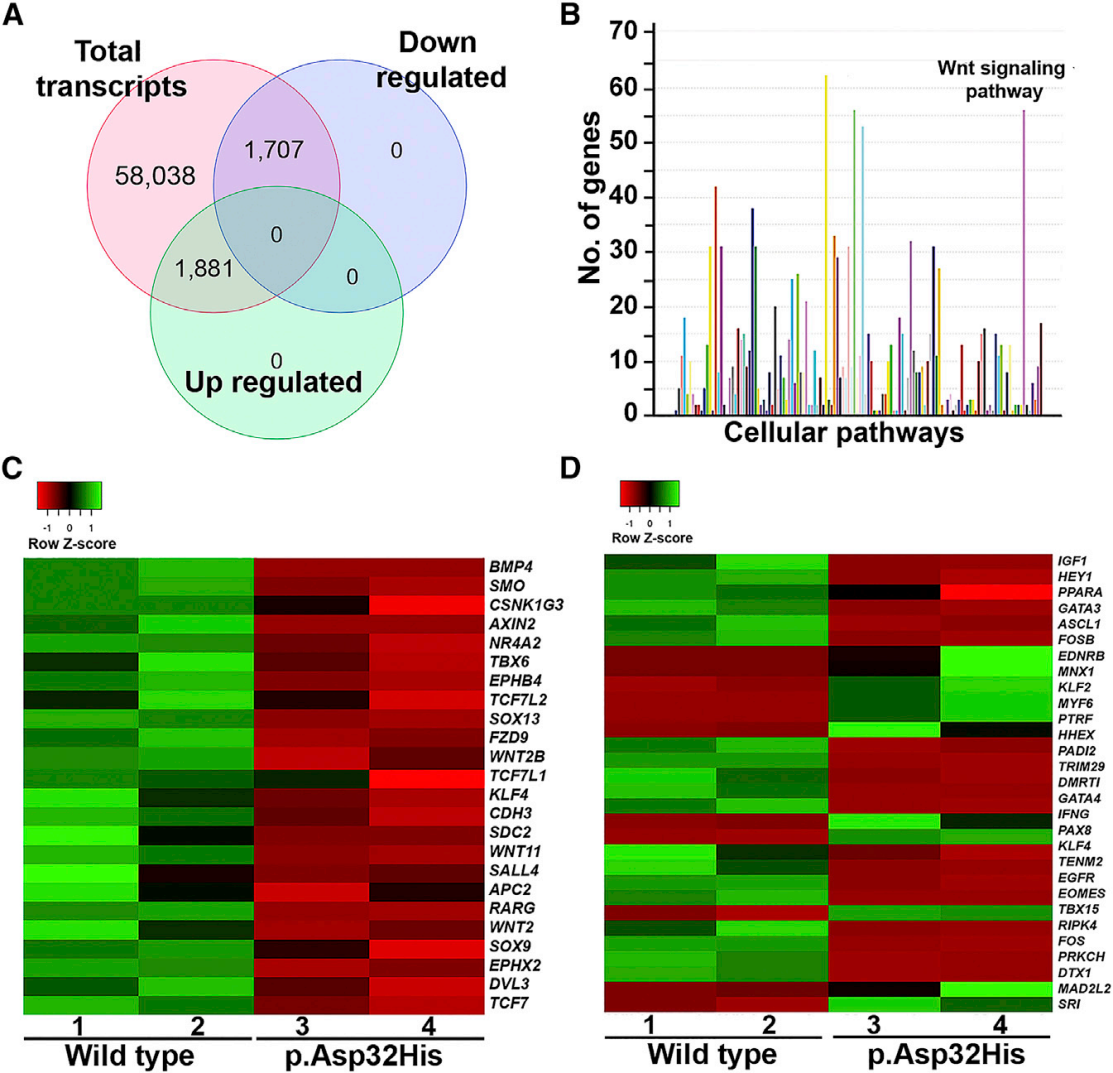
Whole-transcriptome profiling of patient LCLs (NP_001311.3; p.Asp32His) (A) Venn diagram showing total number of transcripts and those differentially regulated. Note that the fold change (FC) > 1, p < 1 for 58,038 transcripts and FC > 2, p < 0.05 for upregulated and downregulated transcripts. (B) Graph showing pathway enrichment of differentially expressed genes obtained from RNA sequencing (RNA-seq) data performed in NP_001311.3; p.Asp32His mutant LCLs and age- and sex-matched control. Note that two of the highest peaks are related to the genes involved in the immune response pathway and Wnt signaling. x axis shows the number of genes, and y axis shows cellular pathways. FC > 5 and p < 0.05. (C) Heatmaps of differentially expressed Wnt target genes observed in NP_001311.3; p.Asp32His mutant LCLs compared with control. Color key shows the level of upregulation or downregulation: green shows higher expression as compared with bright red. (D) Heatmaps of differentially expressed transcription regulators observed in NP_001311.3; p.Asp32His mutant LCLs compared with control.

To investigate the effects of impaired Wnt signaling on downstream target genes, we performed bulk transcriptome profiling on mutant (NP_001311.3; p.Asp32His) and age-matched control LCLs. We obtained >100 M aligned reads for each wild-type and mutant RNA. Data analysis showed 58,038 DEGs (fold change [FC] > 1, p < 1) in wild-type and subject LCLs (Table S4, sheet 1). Nonetheless, using stringent filtering criteria (FC > 1, p < 0.05), we noted 6,500 protein-coding differentially expressed genes (Table S4, sheet 2). Furthermore, we observed 1,707 DEGs were downregulated (FC > 2, p < 0.05) and 1,881 were upregulated (FC > 2, p < 0.05) in patient LCLs (Figure 6A; Table S4, sheets 3 and 4). Pathway enrichment revealed that a maximum number of DEGs was involved in Wnt signaling and immune response pathways (Figure 6B). Further inspection of Wnt signaling genes (FC > 2, p < 0.05) revealed cadherins, NFAT, and Wnt target genes. Among Wnt target genes, we found significant downregulation of 24 genes—*CDH3* (MIM: 114021), *WNT2* (MIM: 147870), *EPHX2* (MIM: 132811), *SMO* (MIM: 601500), *WNT2B* (MIM: 601968), *KLF4* (MIM: 602253), *NR4A2* (MIM: 601828), *APC2* (MIM: 612034), *AXIN2* (MIM: 604025), *RARG* (MIM: 180190), *WNT11* (MIM: 603699), *WNT2B* (MIM: 601968), *SDC2* (MIM: 142460), *FZD9* (MIM: 601766), *TCF7L1* (MIM: 604652), *TCF7L2* (MIM: 602228), *TCF7* (MIM: 189908), *EPHB4* (MIM: 600011), *BMP4* (MIM: 112262), *SOX9* (MIM: 608160), *SOX13* (MIM: 604748), *SALL4* (MIM: 607343), and *TBX6* (MIM: 602427)—including *DVL3* (MIM: 601368) (Figure 6C). The majority of these genes, especially *CDH3*, *TCF7*, and *TCF7L2*, are considered indispensable for normal brain functions.^56^^,^^57^ Pathogenic variants of *BMP4* have been reported to cause brain malformation, digital anomalies (poly/syndactyly), and retinal dystrophy.^58^ Similarly, variants of *SOX9* also cause campomelic dysplasia characterized by severe short stature.^59^

Interestingly, we also found significant differences in the expression pattern of genes (FC > 2, p < 0.05) that code for transcriptional regulators (Figure 6D). These data also point toward impaired Wnt signaling because β-catenin is a transcriptional co-factor, and its dysregulation could affect transcriptional regulators. The data showed high downregulation of 14 transcriptional regulators—*GATA4* (MIM: 600576), *DMRT1* (MIM: 602424), *FOS* (MIM: 164810), *TENM2* (MIM: 610119), *FOSB* (MIM: 164772), *TRIM29* (MIM: 610658), *EGFR* (MIM: 131550), *PADI2* (MIM: 607935), *HHEX* (MIM: 604420), *PRKCH* (MIM: 605437), *ASCL1* (MIM: 100790), *EOMES* (MIM: 604615), *DTX1* (MIM: 602582), and *RIPK4* (MIM: 605706), where *GATA4* was 50 times downregulated and showed the least expression among listed transcription regulators. In contrast, increased expression levels of *BHLHA15* (MIM: 608606), *TBX15* (MIM: 604127), *KLF2* (MIM: 602016), *MNX1* (MIM: 142994), *CAVIN1* (MIM: 603198), *EDNRB* (MIM: 131244), *PAX8* (MIM: 167415), *IFNG* (MIM: 147570), *TNFSF11* (MIM: 602642), *MYF6* (MIM: 159991), and *HGF* (MIM: 142409) were also observed (Figure 6D).

### Phosphoproteome analysis of mutant LCLs also hints at dysregulation of the Wnt signaling pathway

Our data showed that CK2 tetrameric holoenzyme constituted with mutant (NP_001311.3; p.Asp32His) CK2β is unable to phosphorylate a specific target peptide as well as β-catenin (Figures 4B, 4C, and 5D). To validate these data and to observe the effects of this variant on the phosphorylation potential of other reported and novel CK2 substrates, we subjected patient-derived (NP_001311.3; p.Asp32His) LCLs along with age-matched control for whole-phosphoproteome analysis.

**Figure 7:**
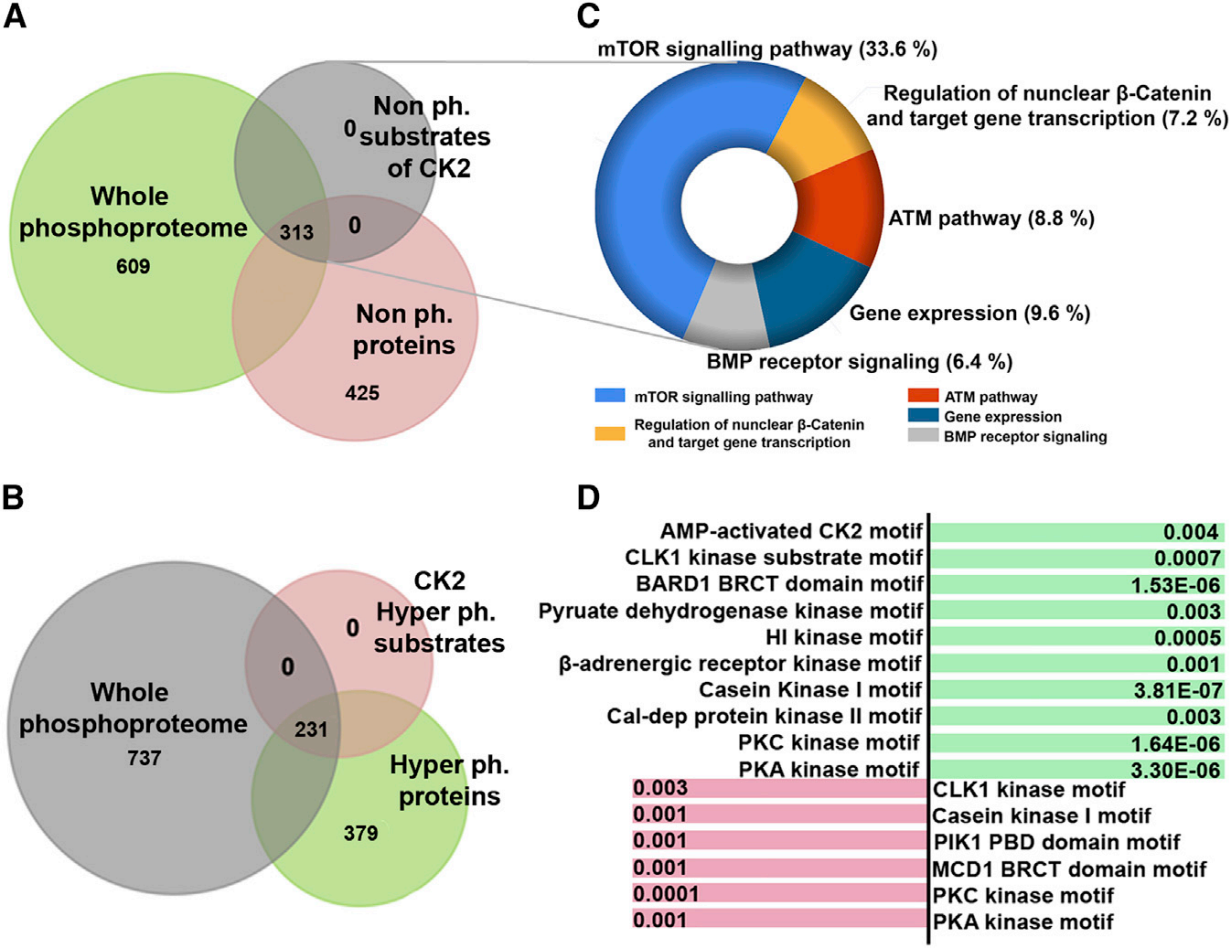
Whole-phosphoproteome profiling of patient LCLs (NP_001311.3; p.Asp32His) (A) Venn diagram showing whole phosphoproteome of mutant (NP_001311.3; p.Asp32His) and wild-type LCLs. Note that among 1,347 (green) proteins, 425 (pink) are non-phosphorylated. These 425 contain 313 putative CK2 substrates motifs (shown in gray). The FC is >2; q value is 0.05. (B) Venn diagram showing whole phosphoproteome of NP_001311.3; p.Asp32His mutant and wild-type LCLs. Note that among 1,347 proteins shown in gray, 379 are hyper-phosphorylated (shown in green). These 379 contain 231 substrates of CK2 (shown in pink). The FC is >2; q value is 0.05. (C) Donut graph generated by Funrich shows the percent of genes involved in a biological pathway. Note that each pathway is color coded and the key is given. (D) The diagram presents substrate motifs of different kinases that are hyper-phosphorylated (shown in green) and hypo-phosphorylated (shown in pink). The respective p values of enriched motifs are written corresponding to each kinase.

Datasets of wild-type and patient-derived (NP_001311.3; p.Asp32His) LCLs were validated by principal-component analysis (PCA) (Figure S6A). We obtained a total of 1,744 motifs of 1,347 proteins (Figure 7A; Table S5). Globally, deregulated phosphorylation events were observed; 425 peptides (754 motifs) were non-phosphorylated (Table S6) and 379 proteins (511 motifs) were hyper-phosphorylated (Figures 7A, 7B, and S6B; Table S7). Intriguingly, out of these 425 non-phosphorylated peptides, 313 (411 motifs) were putative CK2 substrates (Figure 7A; Table S8). Our finding of 313 proteins/substrates lacking phosphorylation events in patient cells corroborated the data of an impaired kinase activity of the CK2 holoenzyme because of the variant NP_001311.3; p.Asp32His of CK2β.

More importantly, pathway enrichment of these 313 non-phosphorylated proteins found in the mutant cells revealed that 7.2% of these proteins (BRCA1, HNRNPA1, CCND2, RANBP9, SKP1, adenomatous polyposis coli [APC], INCENP, and PELP1) play critical roles in the regulation of nuclear β-catenin and transcription of the target genes (Figure 7C). The remaining proteins play vital roles in gene expression, as well as mTOR, ATM, and bone morphogenetic protein (BMP) receptor pathways. When using PANTHER to highlight the most affected cellular processes, these 313 non-phosphorylated proteins were enriched in cellular development and differentiation (Figure S6C).

Notably, among the 313 non-phosphorylated proteins, 72 are known substrates of CK2 (Table S9) either published previously^54^^,^^55^^,^^60^^,^^61^ or found in PhosphoSitePlus v.6.5.9 and Phospho.ELM version 9.0.

Our data also revealed hyper-phosphorylation of 511 motifs of 379 proteins (substrates of β-adrenergic receptor kinase substrates and CK1); out of these, 231 proteins were those that are substrates of calmodulin-dependent and AMP-activated CK2 motifs (Figure 7D).

## Discussion

We present clinical and genetic characterization of five subjects presenting different craniodigital syndromes caused by variants of *CSNK2B*. Deep phenotyping by GestaltMatcher categorized two subjects manifesting POBINDS, while the remaining three subjects were identified presenting a distinct, novel IDCS phenotype. The subphenotypes of craniodigital syndromes in general have been actively debated for whether these represent one single condition or a heterogeneous spectrum.^62^ The clinical characterization of patients with pathogenic variants in *CSNK2B* who display overlap with clinical Filippi syndrome provides evidence toward genetic heterogeneity. Relevantly, phenotypic investigation of a previously reported patient,^8^ carrying our studied variant (GenBank: NM_001320.7:c.94G>A (p.Asp32Asn)), performed through GestaltMatcher, showed striking similarity of facial gestalt compared with subjects 1–3, thus falling into the category of IDCS (Figure S2). Clinical analyses of another reported patient carrying *CSNK2B* variant, GenBank: NM_001320.7:c.94G>T (p.Asp32Tyr), also revealed phenotypic features comparable with subjects 1–3.^24^ These data clue toward possible genotype-phenotype correlation. We also extended our analyses to compare the clinical manifestations of our studied affected members (subjects 1–3) with those reported previously and harboring CK2β: NP_001311.3; p.Phe34Ser and CK2β: NP_001311.3; p.Asn35Lys.^8^ Unfortunately, the published clinical information of both patients was too scarce (lacking information of facial or digital anomalies) to make a differential diagnosis. Therefore, we cannot establish any genotype-phenotype correlation among patients carrying variants in the vicinity of Asp32 of CK2β. Nonetheless, the phenotypic variability observed in the investigated cases could be attributed to the pleiotropic effects of the underlying variants. All three cases of IDCS carried missense variants affecting the same codon of *CSNK2B*. *In silico* tools support the pathogenic nature of the missense variants (Table S1). These inferences are also supported by the fact that *CSNK2B* is highly intolerant to missense (*Z* score = 3.13) and loss-of-function variants (probability of loss of function intolerance [pLI] score = 0.92). Interestingly, both missense variants are located in the KEN box-like motif of CK2β (32-DKFNLTGLN-40), which is known to participate in cell-cycle-dependent protein degradation in other kinases.^63^ The exact role of the KEN box for CK2β has not been characterized yet, but considering its close proximity to the destruction box (attributed for proteasome degradation), it is likely to play a similar role in CK2 as in other kinases. In addition, the CK2β residues from position 20 to 33 are already known to be crucial for its function as an ectokinase.^64^ Interestingly, both variants located in this region might also have an impact on the export of CK2 toward the external cell membrane.

Concerning the nonsense variant NP_001311.3; p.Ser125∗, mutant mRNA is likely to be degraded due to nonsense-mediated decay, which could compromise the catalytic activity of CK2. Moreover, the variant c.367+5delG, identified in subject 5, is predicted to impair the canonical splice donor site (Table S1) and thus likely to cause aberrant splicing. We also explored the effect of this variant on splicing by the minigene splicing assay and observed two different events: the retention and skipping of intron 5 and exon 5, respectively. These findings were exactly similar to consequences of the splice variant c.367+2T>C previously reported to cause POBINDS.^51^ Most of the reported variants of *CSNK2B* in POBINDS patients are protein truncating,^6^^,^^8^^,^^51^^,^^52^ with speculated haploinsufficiency as the underlying pathomechanism.^6^ In line with these observations, we may propose similar consequences of aberrant splicing for variant c.367+5delG and pathogenic haploinsufficiency. In contrast with POBINDS, a dominant-negative effect of the variants identified in our novel IDCS patients could be the underlying mechanism, because the overexpression of CK2β observed in patient cells at both the transcript and protein levels could derive from the mutant version and compromise the activity of the protein encoded by the wild-type allele as well. A previous study has shown that increased expression of CK2 in the fission yeast causes severe growth defects and a multiseptated phenotype.^65^ However, we emphasize the functional characterization of loss-of-function variants identified in affected members manifesting POBINDS can provide further insights into our hypothesis.

*CSNK2B* encodes a polypeptide known as CK2β, which organizes as a stable dimer to regulate the other two catalytic chains (CK2α or CK2α′) of the CK2 holoenzyme, which does not directly bind with one another.^10^ The function of the CK2β dimer within the tetrameric holoenzyme complex has been studied previously. Notably, the C-terminal segment of CK2β (referred to as “CK2α-interacting region” in Figure 2A) is necessary for the interaction with the catalytic CK2 subunits.^10^ It has been reported that protein residues from Asn181 to His193 are fundamental for the regulatory properties of CK2β,^66^ whereas cysteines 109, 114, 137, and 140 of the zinc-finger regions are necessary for assembling the β dimer.^12^ We assume that the missense variants observed in our IDCS patients do not play any role in the dissociation of β dimers because the mutated residue is located distant to the region participating in the formation of the β dimers. Moreover, the identified missense variants are not located in regions previously known to mediate interaction with CK2α. Therefore, we did not observe any effect on the interaction with CK2α even though we used MST that has superior sensitivity than classical techniques such as pull-down and co-immunoprecipitation assays.

A previous study using MS combined with affinity chromatography of mouse brain revealed 144 CK2-independent binding partners of CK2β, out of which 32 are involved in protein formation and degradation.^67^ Our study identified 194 independent binding partners of CK2β, only 47 of which have been previously reported. Our findings thus add to the number of CK2β partners and indicated its expanded role into novel pathways.

**Figure 8:**
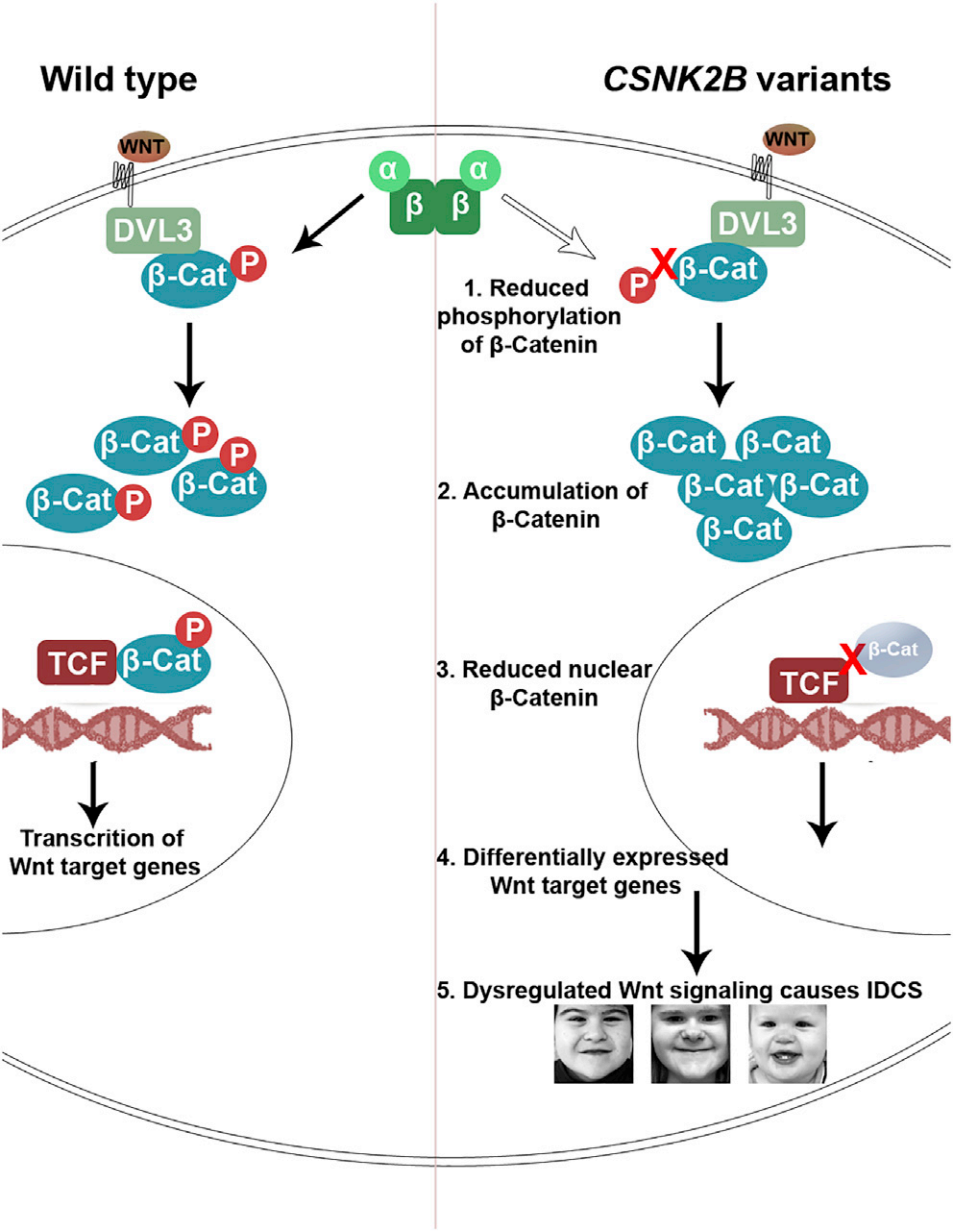
CK2 is the crucial member of the canonical Wnt signaling pathway Mutant CK2 has reduced the phosphorylation rate of β-catenin, which thus fails to translocate to the nucleus and consequently affects the regulation of Wnt target genes. This disruption of Wnt signaling leads to the phenotype seen in the studied patient.

CK2 is known to be involved in the regulation of diverse cellular pathways; the list of its substrates is growing, which makes it challenging to choose any specific pathway to dissect the pathomechanism of a specific disease associated with variants in *CSNK2B*. Interestingly, our protein-protein interactome data of the variant NP_001311.3; p.Asp32His hinted its effect in Wnt signaling, in particular the degradation of β-catenin. The Wnt signaling pathway is well known to orchestrate a variety of cellular processes, especially during development. Any perturbation in Wnt signaling can lead to phenotypes including microcephaly, limb malformations, dermal hypoplasia, and Williams-Beuren syndrome (MIM: 194050).^68^^,^^69^ β-Catenin, a key player of Wnt signaling, forms multiple complexes that control cell proliferation. In case of negative regulation (when Wnt signaling is off), the cytoplasmic β-catenin is associated with the destruction complex (Axin, APC, kinases GSK-3α/β and CK1) and undergoes proteasomal degradation.^70^ However, when Wnt signaling is on, the destruction complex is dismissed, and free β-catenin, after being phosphorylated by CK2, forms a complex with DVL3; this CK2/Dvl/β-catenin complex allows β-catenin to be transported into the nucleus, where it binds to high-mobility group box transcription factors of the TCF/LEF family and initiates transcription of target genes chiefly involved in growth and development (Figure 8).^15^ Phosphorylation of β-catenin by CK2 maintains its stabilization and translocation into the nucleus to serve as a cofactor for transcription.^71^ Previous studies also suggest that if CK2 phosphorylation is inhibited, the cytoplasmic/nuclear ratio of β-catenin is altered and β-catenin can no longer perform its transcriptional activities.^15^ These observations prompted us to investigate Wnt signaling in our patients.

To assay possible dysregulation of Wnt signaling, we investigated the expression of β-catenin in both patient LCLs harboring NP_001311.3; p.Asp32His and NP_001311.3; p.Asp32Asn variants. We found that the active/nuclear β-catenin was drastically reduced in the patient LCLs. These findings suggest that the CK2-mediated phosphorylation may be impaired because of a reduced kinase activity of CK2 containing a mutated β subunit. Our analysis for the kinase activity of CK2 with both variant beta subunit NP_001311.3; p.Asp32His and NP_001311.3; p.Asp32Asn for β-catenin as their substrates indicated a significant reduction of kinase activity. These data suggest that β-catenin may be a class III substrate or at least a class I substrate of CK2.

Interestingly, the X-ray crystallography of CK2 holoenzyme also indicated its impaired kinase activity when containing the variant CK2β subunits because the mutated residue (Asp32) is in the vicinity of the ATP binding loop of the neighboring CK2α subunit. There, suitable substrate peptides and proteins are primarily recognized by the sequence environment around the Ser/Thr side chain for phosphorylation (position p0 in Figure 2D). To this end, CK2α exposes a number of positively charged residues: in particular, Lys198 and Arg80, which coordinate with negatively charged side chains at the p+1 and the p+3 positions, respectively, of CK2 substrates (Figure 2D). Furthermore, Arg80 is also a part of an extended “basic cluster” at the beginning of helix αC of CK2α (Figure 2D): the four lysine side chains of this positively charged patch contribute to substrate recognition because acidic residues beyond the p+3 position of CK2 substrates support phosphorylation as well.^72^

CK2β stimulates the catalytic activity of CK2α as long as small peptides and so-called class I proteins (e.g., casein) serve as substrates. A specific function of Asp32 in this context was, to our knowledge, never described. However, the close proximity of Asp32 to the substrate recognition region of CK2α within the CK2α2β2 holoenzyme suggests a significant role of Asp32 in recognizing the CK2 substrates (Figure 2D). It is possible that the negative charge of Asp32 of CK2β supports the release of a likewise negatively charged phosphopeptide. If this is true, which remains to be experimentally shown, an impact of a loss of the negative charge (variant Asp32Asn) or even its conversion into a positive one (variant Asp32His) appears to be plausible.

The kinase activity analyzed using a target peptide of CK2 showed that only the variant NP_001311.3; p.Asp32His compromised the kinase activity of CK2, but not the variant NP_001311.3; p.Asp32Asn. We speculate that the latter may have milder effects on the efficiency of CK2 and still be able to phosphorylate subsets of substrates. This would also explain the milder phenotype of the NP_001311.3; p.Asp32Asn patient as compared with the patient with the variant NP_001311.3; p.Asp32His. It is also likely that the reduced kinase activity could be because of the variable impact of both variants on the structural integrity and thus the interaction of both mutant beta subunits with its binding partners or substrate recognition. Furthermore, it was evident from our findings that DVL3 also showed reduced interaction with mutant CK2β, thereby suggesting an impairment in the formation of CK2β/DVL3/β-catenin complex and thus impairing the whole positive regulation of Wnt signaling.

Because our data revealed a more severe effect of NP_001311.3; p.Asp32His on the kinase activity of CK2 as compared with NP_001311.3; p.Asp32Asn, we performed whole-phosphoproteome profiling only in LCLs derived from the patient harboring variant NP_001311.3; p.Asp32His. Intriguingly, it showed a loss of phosphorylation for 313 putative substrates of CK2. *In silico* functional enrichment of these proteins clearly showed their role in the regulation of nuclear β-catenin and expression of target genes. These results further strengthen and validate our findings of reduced kinase activity of CK2 containing variant beta subunits. The effect on nuclear or active β-catenin is critical because it functions as a transcriptional co-activator and participates in diverse cellular pathways by positive and negative regulatory mechanisms of Wnt signaling.^70^ This regulation is critical for maintaining the balance of β-catenin, thus eventually controlling the orchestration of transcriptional activities. Additionally, we have also seen a loss of phosphorylation for APC and DVL3 in patient cells, which further corroborates our finding of an impaired Wnt signaling. With special reference to calmodulin-dependent CK2 (CaMKII), a drastic hyperphosphorylation activity was observed that is an expected consequence because CK2β, in this case, works as an inhibitor of CK2α to control the normal activity of calmodulin.^73^ In addition, it has already been reported that genetically altered mice overexpressing calmodulin develop severe cardiac conditions caused by increased autonomous activity of CaMKII *in vivo*.^74^ This observation is in line with manifestation of heart anomaly observed in patient 1. The deregulated phosphorylation of protein kinase A, protein kinase C, and β-adrenergic receptor kinases also suggest abnormalities in the signal transduction in patient cells causing neurological conditions.^75^ Conclusively, phosphoproteome data strongly support the impaired phosphorylation capacity of CK2 caused by a variant in CK2β. It also extends the knowledge about deregulation of other kinases in the patient LCLs.

In the light of these findings, we suggest that CK2β might specifically mediate the nuclear translocation of β-catenin. Deregulation in kinase activity of free variant CK2β or CK2 containing a variant beta subunit may cause an impairment of β-catenin-dependent Wnt signaling, which affects the transcription of downstream target genes. The hypothesis was supported by gene expression at both transcriptome and proteome profiling of patient LCLs. In particular, the expression of well-established proteins of the Wnt signaling pathway was similar and in line with the results at transcriptional level. Surprisingly, an elevated differential expression of genes involved in the immune system was also observed in the patient’s transcriptome, which prompted us to review the phenotypic profile of the patient for finding a link between differential expression of immune response genes and patient disease conditions. For this purpose, a blood profile of the patient was obtained, which revealed comparatively high levels of IgM (immunoglobulin M): 253 mg/dL as compared with a normal range of 40–230 mg/dL. The only infection noticed during clinical investigation was recurrent otitis, which was supposed to be due to a stenosis of the duct.

Based on all these findings, we conclude that the identified pathogenic missense variants in *CSNK2B* cause pathogenic accumulation of CK2β that dysregulates the Wnt signaling pathway. Due to an impaired phosphorylation activity of variant CK2 and a loss of interaction with crucial regulators of Wnt signaling, regulation of active β-catenin is most likely impaired, leading to altered transcription resulting in the observed clinical phenotype.

## Consortia

The members of the Italian Undiagnosed Diseases Network are Domenica Taruscio, Marco Salvatore, Agata Polizzi, Federica Censi, Giovanna Floridia, Giuseppe Novelli, Erica Daina, Alessandra Ferlini, Marcella Neri, Dario Roccatello, Simone Baldovino, and Elisa Menegatti.

## Web resources

UniProt, https://www.uniprot.org/

National Center for Biotechnology Information, https://www.ncbi.nlm.nih.gov/

PANTHER Classification System, http://www.pantherdb.org/

Predict Protein, https://www.predictprotein.org/

IUPred3, http://iupred.enzim.hu/

FoldIndex, http://bip.weizmann.ac.il/fldbin/findex

PhosphoSitePlus, https://www.phosphosite.org/homeAction

Phospho.ELM, http://phospho.elm.eu.org/dataset.html

OMIM, http://www.omim.org

### Data and code availability

The investigated variants have been submitted to ClinVar with the following accession numbers: SCV002498742, SCV002498743, VCV000520596, SCV002498744, and SCV002498745. The proteome/phosphoproteome data generated during this study are available at Gene Expression Omnibus and ProteomeXchange consortium with accession numbers GEO: GSE189065 and PXD029970 and PXD029983, respectively. The remaining associated data for this study can be provided on reasonable request.

## Supplemental information

Document S1. Figures S1–S6, Tables S1–S3 and S9, and Supplemental material and methods

Table S4. List of differentially expressed genes found in transcriptome data

Table S5. List of phosphoproteome found in wild-type and patient LCLs

Table S6. List of nonphosphorylated motifs observed in patient LCLs

Table S7. List of hyper-phosphorylated motifs observed in patient LCLs

Table S8. Putative CK2 target motifs found non-phosphorylated in the patient LCLs

Document S2. Article plus supplemental information
