# Supplementary material for: De novo variants of CSNK2B cause a new intellectual disability-craniodigital syndrome by disrupting the canonical Wnt signaling pathway
Source: HGG Adv. 2022 Apr 18;3(3):100111. doi: 10.1016/j.xhgg.2022.100111 (PMC9092267; doi:10.1016/j.xhgg.2022.100111)
Supplement: Document S1. Figures S1–S6, Tables S1–S3 and S9, and Supplemental material and methods [file mmc1.pdf]

HGGA, Volume *3*

Supplemental information

De novo variants of *CSNK2B* cause a new intellectual disability-craniodigital syndrome by disrupting the canonical Wnt signaling pathway

Maria Asif, Emrah Kaygusuz, Marwan Shinawi, Anna Nickelsen, Tzung-Chien Hsieh, Prerana Wagle, Birgit S. Budde, Jennifer Hochscherf, Uzma Abdullah, Stefan Höning, Christian Nienberg, Dirk Lindenblatt, Angelika A. Noegel, Janine Altmüller, Holger Thiele, Susanne Motameny, Nicole Fleischer, Idan Segal, Lynn Pais, Sigrid Tinschert, Nadra Nasser Samra, Juliann M. Savatt, Natasha L. Rudy, Chiara De Luca, Italian Undiagnosed Diseases Network, Paola Fortugno, Susan M. White, Peter Krawitz, Anna C.E. Hurst, Karsten Niefind, Joachim Jose, Francesco Brancati, Peter Nürnberg, and Muhammad Sajid Hussain

Supplemental Data

Supplemental data include clinical details of POBINDS patients, six figures, nine tables, supplemental methods and references.

Clinical synopsis of POBINDS and IDCS Patients

Patient 4 of our cohort is recruited from GeneMatcher is a 6-year-old male from Israel (Figure 1). He presented phenotypes including prominent facial dysmorphic features including, long face (HP:0000276), protruding ears (HP:0000411), high forehead (HP:0000348), broad nasal bridge (HP:0000431) and hair hypopigmentation (HP:0005599) (center of head). In addition, low muscle tone (HP:0001252) and ligament laxity (HP:0001388) was also noted. Regarding intellectual abilities , intelligence quotient was noted around 70 which denote mild intellectual disability (HP:0001249). Additionally, low speech intelligibility, learning disability (HP:0001328), emotional regulation disorder, short attention span (HP:0000736) and low verbal performance were also recorded (Table 1).

Electroencephalogram showed centro temporal spike waves signifying epilepsy (HP:0001250). Carnitine level in patient's blood test was higher than normal i.e., 42.3 micro mol/L suggesting mild cardial defect. On the other hand, the patient did not show any symptoms of craniodigital or teeth anomaly. MRI showed normal brain morphology (data not shown).

Patient 5 of our cohort was identified via GeneMatcher. The patient's data was submitted to GeneMatcher on behalf of the family after they chose to participate in GenomeConnect, the ClinGen patient registry.[1] Patient 5 (Figure 1) was a 30-year-old American female who died of epileptic seizures (HP:0001250). The patient was reported to have seziures at the age of 1 year. Seizure types included tonic-clonic (HP:0002069), myoclonic (HP:0032794), clonic (HP:0020221), absence seizures (HP:0002121). Moreover, she was evaluated by the clinician at the age of 15 months for possible variant of Maple Syrup Urine disease (MIM#248600) as intermittent "sweet" body odor was noticed. Her physical examination showed a weight of 10.2 kg (25th %ile), a height of 78 cm (25th %ile), and a head circumference of 47 cm (50th %ile). Other phenotypic features include eye movement issues, myopia (HP:0000545), hyperopia (HP:0000540), borderline intellectual functioning (IQ (WASI II) = 67, SS (WRAT IV) = 82), memory impairment (HP:0002354), anxiety (HP:0000739), depression (HP:0000716), obsessive compulsive disorder (HP:0000722), low muscle tone (HP:0001252), proprioception issues (HP:0010831), and prognathism

(HP:0000303) with pointed chin (HP:0000307). Her general physical examination was unremarkable, except for mild generalized hypotonia (HP:0001290) and increased range of motion to her major joints corresponding to her motor tone. She presented no major dysmorphic features except flat feet.

Metabolic evaluation noted very mildly elevated plasma branched chain amino acids, but no elevation in allolleucine. Subsequent skin fibroblast studies in Dean Danner's lab at Emory were normal for the branched-chain ketoacid dehydrogenase complex. Also, urine organic acids were normal.

Patient 7

Individual 7 is a 13-year-old son of healthy unrelated Australian parents, reported previously.[2] He was born at term with birth parameters of weight 3.15 kg, length 48 cm and head circumference 34 cm. He was noted to have a cleft soft palate (HP:0000185) and a patent ductus arteriosus (HP:0001643) and unilateral renal hypoplasia (HPO:0008678). He proceeded to have hypotonia (HP:0001290) and developmental delay and was diagnosed with mild to moderate intellectual disability (HP:0001249) with an IQ of 53 and developmental dyspraxia. He developed absence epilepsy requiring anticonvulsant treatment. He had structurally normal eyes. He had recurrent otitis media and had tympanostomy tube insertion.

Growth at age 13 years showed weight on the 95th centile and height on the 19th centile and head circumference 55th centile. He had anteverted ears (HP:0040080), upslanted palpebral fissures (HP:0000582), bulbous nose (HP:0000414), smooth philtrum (HP:0000319) and thin upper lip vermilion (HP:0000219). He had a supernumerary tooth (HP:0011069) near the midline between his two central incisors. He had brachydactyly (HP:0001156), spatulate fingers and down-sloping shoulders (HP:0200021). Brain MRI peformed at the age of 5 years was unremarkable.

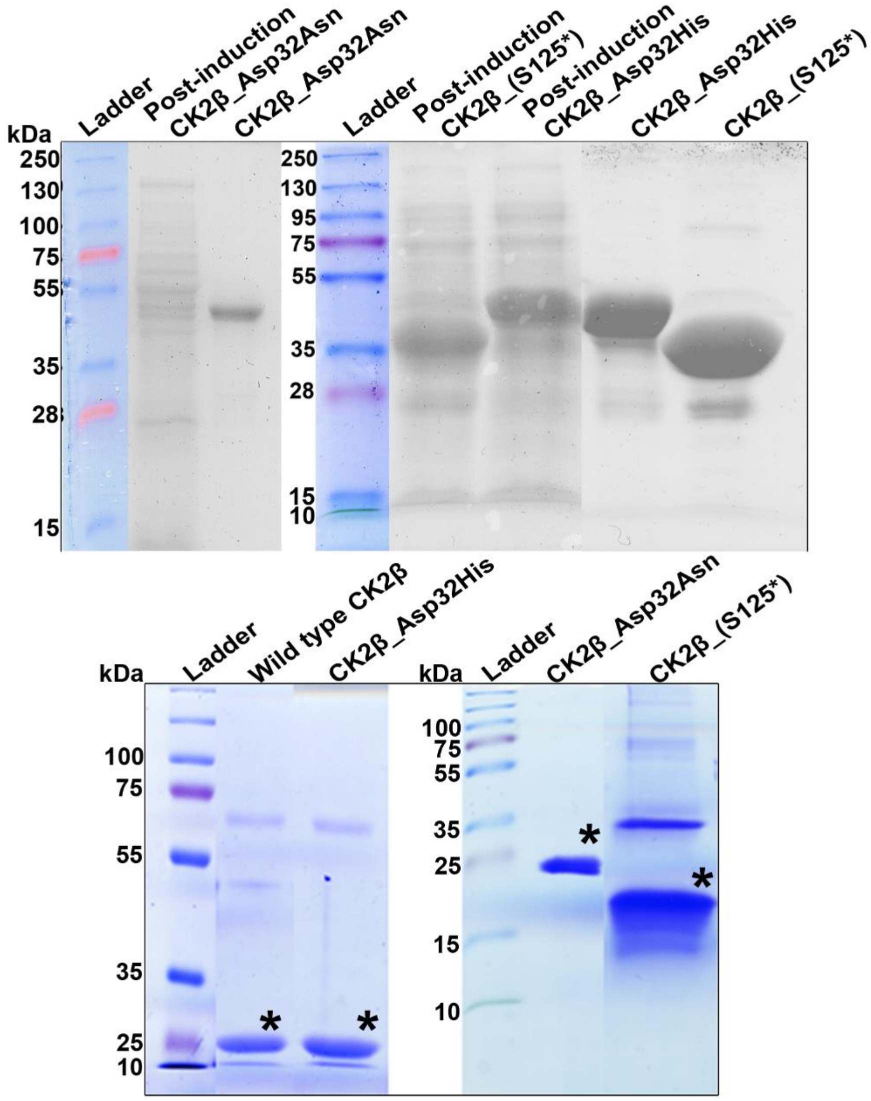

Figure S1. Purification of GST fused CK2β and obtaining the cleaved protein product

(A) Coomassie stained SDS-PAGE gel showing purified GST tagged wild-type CK2β and its mutants (p.Asp32His, p.Asp32Asn and p.Ser125*). Note: Image is obtained by two different gels.

(B) SDS-PAGE showing purified cleaved protein of wild-type CK2β and its mutants (p.Asp32His, p.Asp32Asn and p.Ser125*) indicated by asterisks. Note: Image is obtained by two different gels.

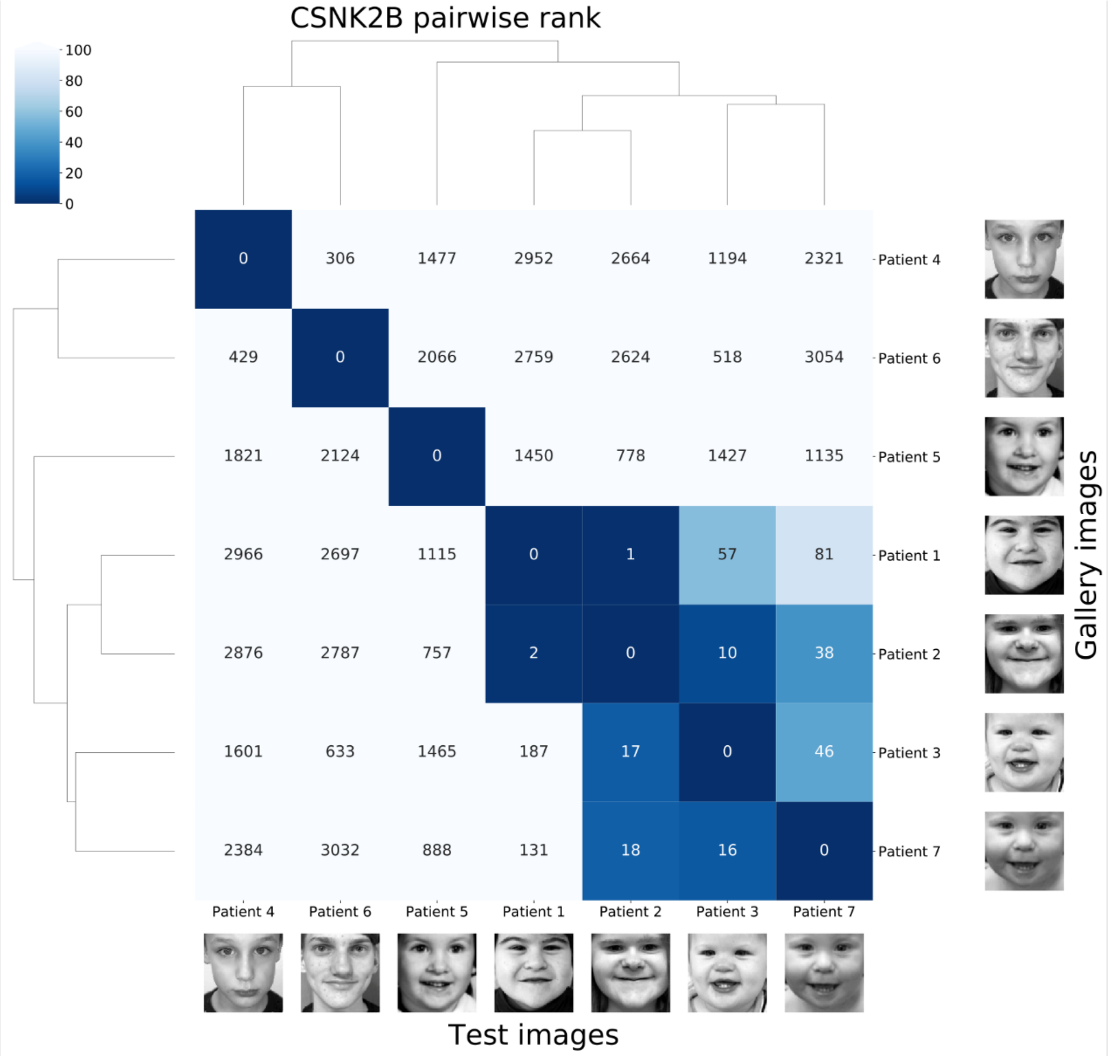

Figure S2. Comparisons of IDCS and POBINDS patients by GestaltMatcher

This figure shows the pairwise rank and hierarchical clustering of seven photos in CFPS. Gallery images were the images to be matched in CFPS. Each column is the result of testing one subject in the column and listing the rank of the rest six photos in each row. For example, by testing Patient 2, Patient 1 was on the 1st rank, and Patient 3 was on the 17th rank of Patient 2. Notably, to avoid bias due to the age difference, we have used the photo of Patient 5 at a younger age instead of the photo shown in Figure 1.

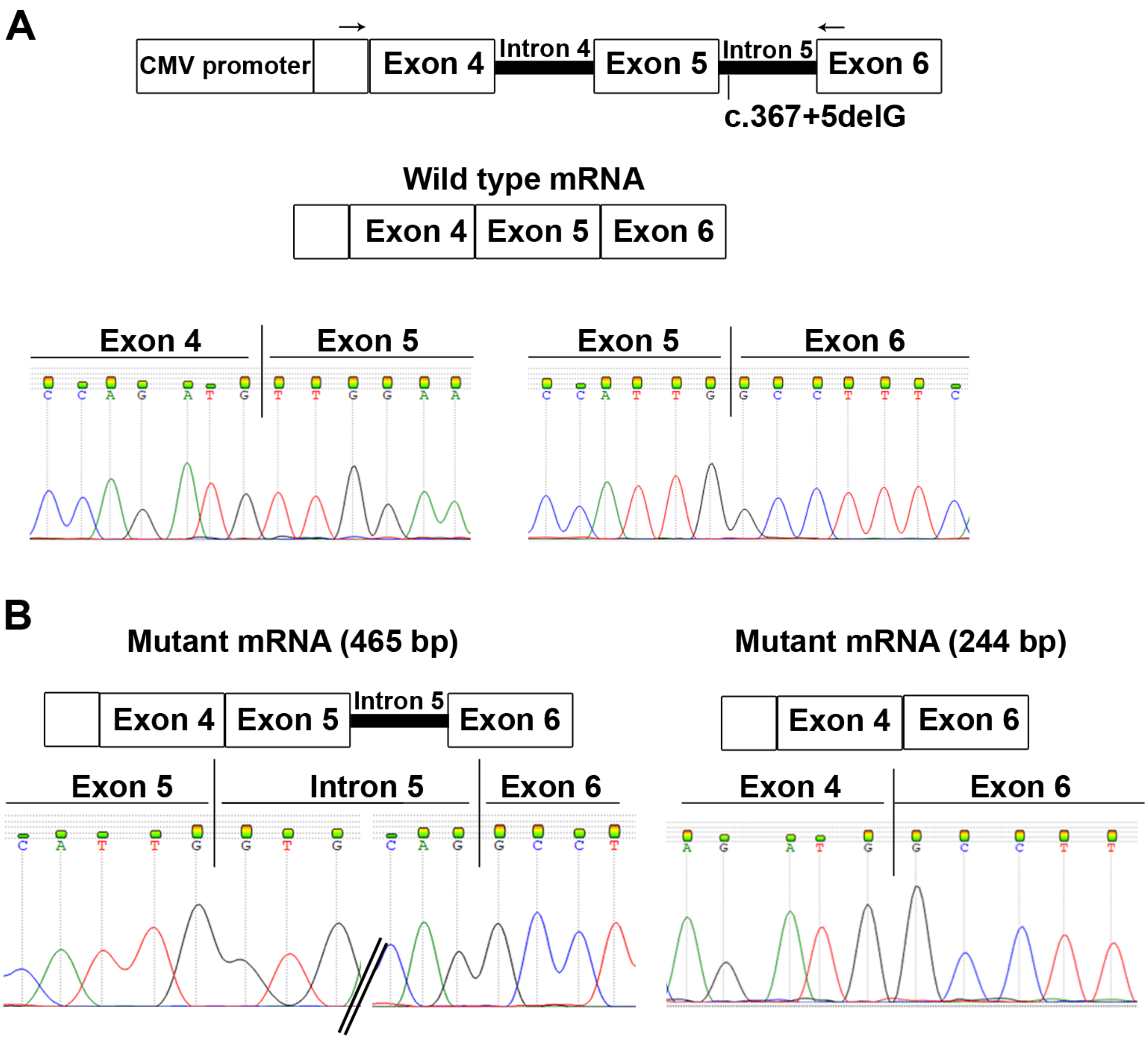

Figure S3. Minigene splicing assay and Sanger sequencing of the RT-PCR products.

(A) Upper panel, schematic of part of the mammalian expression vector, pCMV-3Tag-3a and insert. Complete exon 4, 5 and 6, along with intron 4 and 5 of *CSNK2B* (ENST00000375882.7;CSNK2B-203) were cloned. Arrows show the locations of forward (located within the vector) and reverse (situated within exon 6) primers used to amplify the transcribed product. Middle panel, schematic of wild-type transcript. Lower panel, Sanger traces of right side showing the junctions of exon 4 and 5, whereas left side indicate the boundary of exon 5 and 6.

(B) Left panel, schematic (on top) and Sanger traces (in the bottom) of the mutant transcript where complete intron 5 was retained. Right panel, schematic (on top) and Sanger traces (in the bottom) of the mutant transcript where complete exon 5 is skipped.

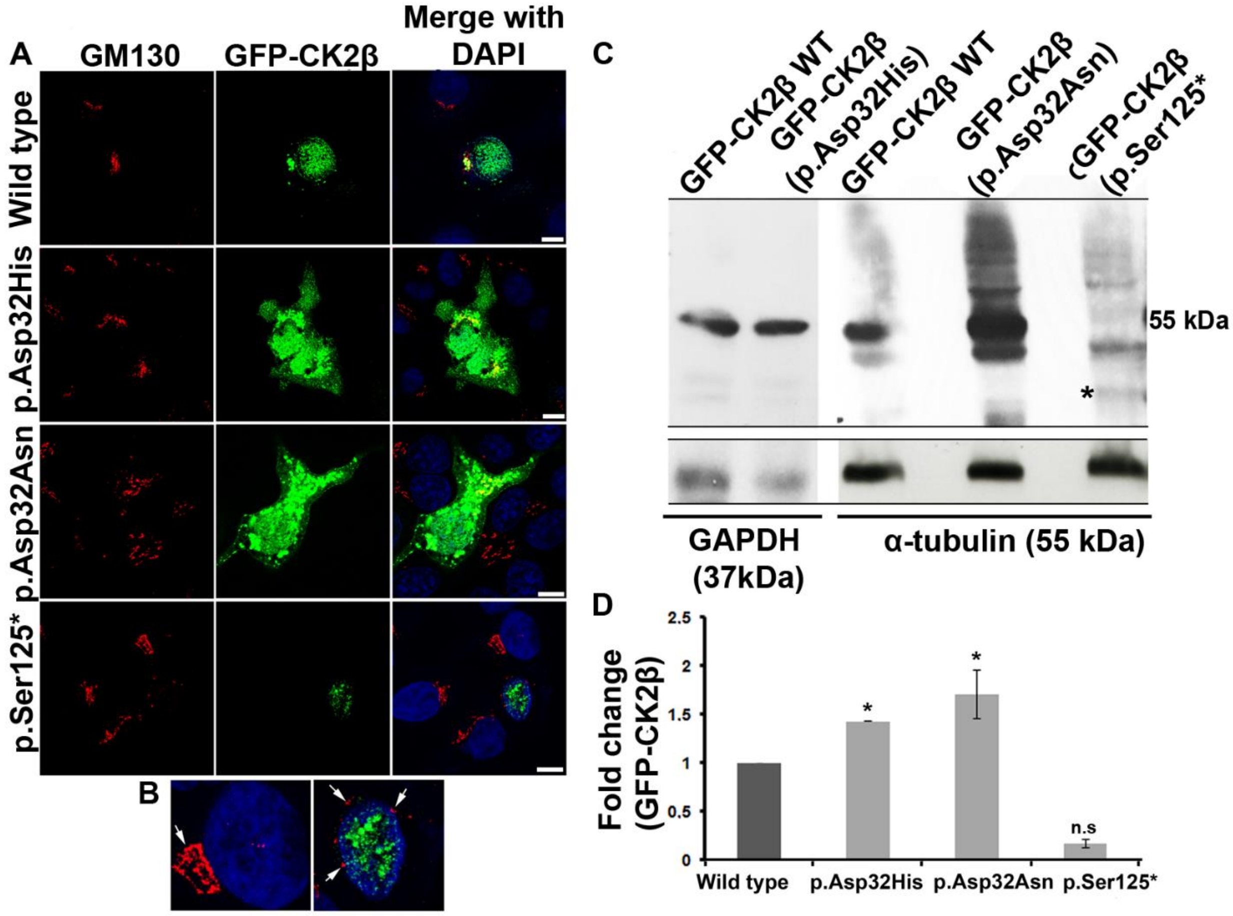

Figure S4. Localization of GFP tagged wild-type and mutants CK2β

(A) Confocal microscopy images showing mislocalized mutant GFP tagged CK2β as compared to wild type in HeLa cells. Scale bar is 10 μm.

(B) CK2β mutant (p.Ser125*), shown at the right hand side, revealing abnormal morphology of Golgi apparatus pointed by white arrows as compared to wild type cell shown at the left hand side.

(C) Immunoblotting shows GFP tagged CK2β on 55 kDa band size in wild type and two mutants (p.Asp32His, p.Asp32Asn). Note that loading control is α-tubulin (55 kDa) except for p.Asp32His, where GAPDH, 37 kDa, was used for loading control. Asterisk (*) shows the expected band of truncated protein (p.Ser125*).

(D) Graph showing the fold change of GFP tagged CK2β wild type in comparison with all three mutants, p.Asp32His, p.Asp32Asn and p.Ser125*. Note: n = 2 and asterisk (*) shows p values of 0.05 (Student's *t* test). Error bar shows standard deviation.

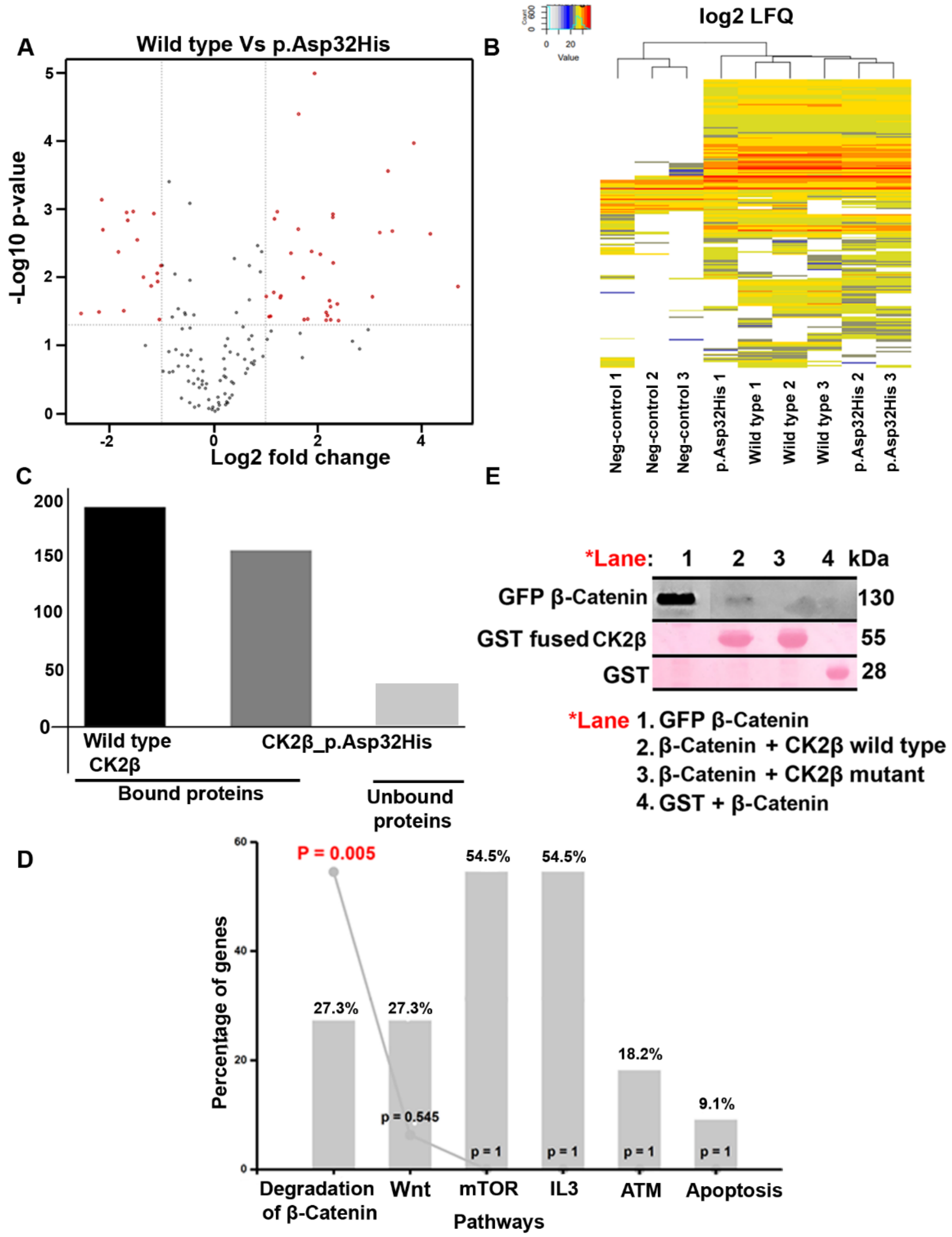

Figure S5. Mass spectrometry data of wild-type and mutant, p.Asp32His, CK2β

(A) Volcano plot interaction of protein binding partners of CK2β wild type and mutant (p.Asp32His).

(B) Heatmap showing the interaction of binding pratners of CK2β wild type and mutant (p.Asp32His). Please notice that there are three replicates of each sample and binding affinity is shown by the color scheme given in the figure.

(C) Graph shows total number of proteins (194) detected in mass spectrometry data. 156 proteins showed interaction whereas 38 proteins showed no interaction with mutant GST tagged CK2β as compared to wild type.

(D) Pathway enrichment of proteins showing compromised interaction with mutant CK2β. Specific pathway is written below each bar.

(E) Pull-down assay indicates reduced interaction of over expressed GFP tagged β-catenin with mutant GST tagged CK2β as compared to wild type in HeLa cells. GFP antibody was used to detect GFP-fused proteins. Membrane was stained with Ponceau S to observe the equal amount of GST tagged proteins used in this assay. GST serves as negative control. Key of lane (red with asterisk) is shown below in the figure.

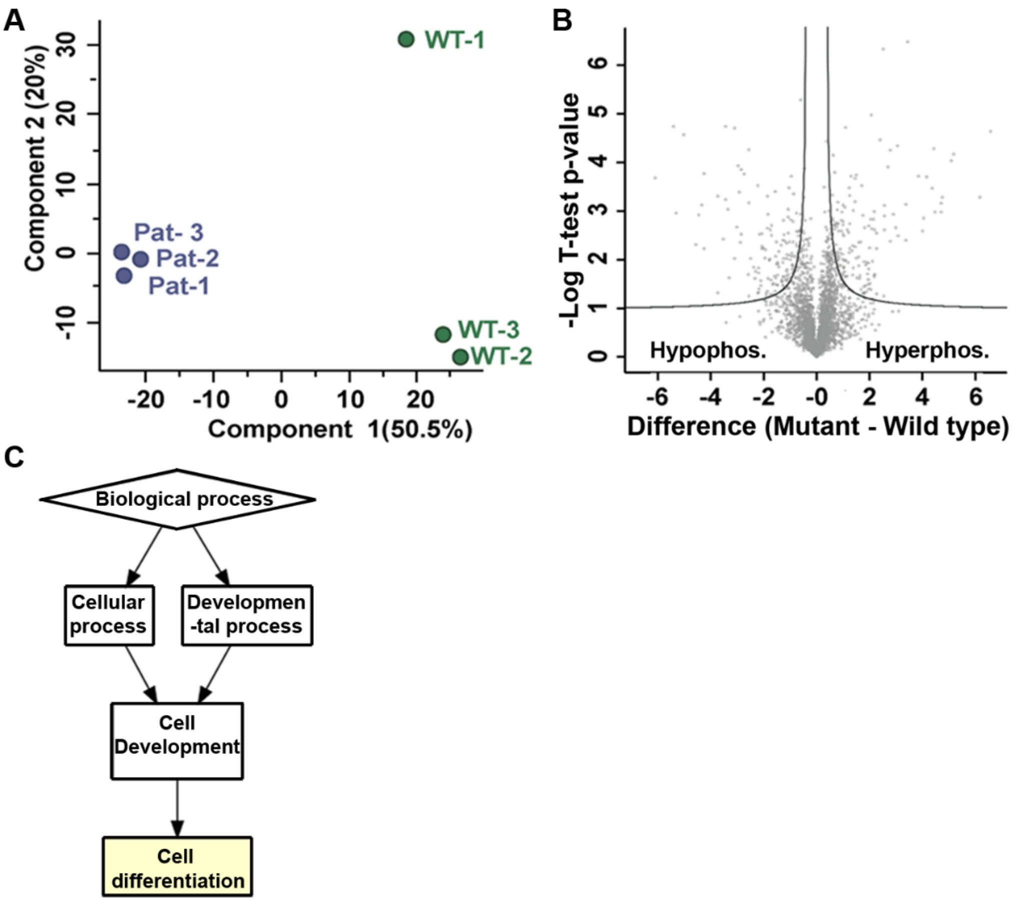

Figure S6. Phosphoproteome analysis of wild-type and mutant LCLs (p.Asp32His).

(A) PCA of three replicates of wild-type and mutant samples submitted for phosphoproteome.

(B) Volcano plot of proteins enriched in phosphoproteome.

(C) Graph generated by GOrilla shows pathway enrichment of those protein peptides which showed no phosphorylation in case of p.Asp32His mutant CK2β. Y-axis shows the percentage of the protein involved in specific pathway shown on the X-axis

Supplemental Tables

Table S1. Pathogenicity scores of *CSNK2B* variants predicted by various in silico tools

Patient ID	Gene/ Transcript ID	cDNA variant	Protein variant	Inheritance/allele frequency gnomAD	pLI	Z-score	CADD score
Patient **1**	*CSNK2B*/ NM_001320.7	c.94G>C	p.Asp32His	*De novo*/0	pLI = 0.92 o/e = 0.08	Z = 3.13 o/e = 0.21	31
Patient **2**		c.94G>A (rs1554169984)	p.Asp32Asn	*De novo*/0			32
Patient **3**		c.94G>A (rs1554169984)	p.Asp32Asn	Unknown/0			32
Patient **4**		c.374C>G	p.Ser125*	*De novo*/0			39
Patient **5**		c.367+5delG (rs1583610622)	p.?	Unknown/0			**na**

Prediction at protein level

Prediction tool	Function	Prediction			
		**p.Asp32His**	**p.Asp32Asn**	**p.Ser125***	**c.367+5delG**
**ACMG interpretation**		Likely pathogenic (PM1, PM2, PM5, PP3, BP1)	Uncertain Significance (PM1, PM2, PP3, BP1)	Pathogenic (PVS1, PM2, PP3)	-
**Mutation Taster**		Disease causing (81)	Disease causing (23)	Disease causing (6.0)	-
**Polyphen-2**		Probably damaging (1.0)	Probably damaging (1.0)	-	-
**PROVEAN**	Protein variation effect analyzer	Deleterious	Deleterious	Deleterious	-
**SNAP^2**	Functional effect classifier on the basis of neural networks	Pathogenic	Pathogenic	-	-
**SNAP&GO**	Variant effect prediction using			-	-

	gene ontology terms	Disease causing	Disease causing		
**PANTHER**	Protein analysis through evolutionary Relationships	Disease causing (0.9)	Disease causing (0.9)	-	-
**PhD-SNP**	Predictor of human deleterious single nucleotide polymorphisms	Disease causing (0.8)	Disease causing (0.8)	-	-
**SIFT**	Predict effects of nonsynonymous / missense variants	Disease causing (0.03)	Disease causing (0.03)	-	-
**SNAP**	Prediction of nonsynonymous functional effects	Disease causing (0.6)	Disease causing (0.6)	-	-
**Meta-SNP**	Meta-predictor of disease causing variants	Disease causing (0.74)	Disease causing (0.74)	-	-
**MUpro**	Prediction of Protein Stability Changes for Single Site Mutations from Sequences	Decreased -1.2239963	Decreased -1.045697	-	-
**I-Mutant**		Decreased (DDG=0.41, **°C=**25)	Decreased (DDG=-0.53, **°C=**25)	-	-
		Increased (DDG=0.30, **°C=**60)	Increased (DDG=0.16, **°C=**25)		-
		Increased (DDG=0.65, **°C=**80)	Increased (DDG=0.50, **°C=**25)		-
**NetGene2 Server**	-	-	-	-	Loss of splice donor site
**ASSP**	Alternative Splice Site Predictor				Loss of constitutive donor site

**MaxEntScan::score5ss**	For human 5' splice sites				**WT**: MAXENT:9.27, MDD: 13.68, MM: 8.22, WMM: 7.62<br>**MUT**: MAXENT: 2.11, MDD: 2.78, MM: 3.19, WMM: 3.71

Loss-of-function intolerant (pLI), delta delta G (DDG), combined annotation dependent depletion (CADD) and Alternative Splice Site Predictor (ASSP). Note: Values given in the parentheses indicate the scores obtained from respective tool.

Table S2. List of oligonucleotides used for co-segregation, site-directed mutagenesis and qPCR

**Primer ID**	**Forward primer, 5' to 3'**	**Reverse primer, 5' to 3'**
**Oligonucleotides sequences for co-segregation analysis**		
CSNK2B-Ex2-F	GGTGAACTAGGGAGAGACACAAG	GCATGAATTTGGGGTTAAAGA
**Oligonucleotides sequences for site-directed mutagenesis**		
*CSNK2B;*c.94G>C	GTGGATGAAGACTACATCCAGCACAAATTTAATCTTACTGGAC	GTCCAGTAAGATTAAATTTGTGCTGGATGTAGTCTTCATCCAC
*CSNK2B;*c.94G>A	GTGGATGAAGACTACATCCAGAACAAATTTAATCTTACTGGAC	GTCCAGTAAGATTAAATTTGTTCTGGATGTAGTCTTCATCCAC
*CSNK2B;*c.374C>G	GCTTCCCATTGGCCTTTGAGACATCCCAGG	CCTGGGATGTCTCAAAGGCCAATGGGAAGC
**Oligonucleotides sequences for quantitative real time PCR**		
*CSNK2B;*c.94G>A	TCTTCTGTGAAGTGGATGAAGAC	CCTTGCTGGTACTTTTCCAAC
*CSNK2B;*c.94G>A	GTGAAGCCATGGTGAAGCTC	GACTGGGCTCTTGAAGTTGC
GAPDH	TGACAACAGCCTCAAGATCATCAGCAA	GTTTTTCTAGACGGCAGGTCAGGTCCA
**Oligonucleotides sequences for sequencing RT-PCR product obtained from minigene splicing assay**		

Vector+CSNK2B	TGAACCGTCAGATCCGCTAG	GCACTTGGGGCAGTAGAGC

Table S3. List of proteins identified in mass spectrometry analysis.

Sr. No	Gene names	Protein names	Status	Interaction
Cell Adhesion Proteins				
1	*DSC1*	Desmocollin-1	Known	No effect
**2**	***PNN***	**Pinin**	**Known**	**Impaired**
3	*DSG1*	Desmoglein-1	Known	No effect
Chaperons				
4	*TOR4A*	Torsin-4A	Novel	No effect
Chromatin-related				
5	*SRCAP*	Helicase SRCAP		No effect
**6**	***ACIN1***	**Apoptotic chromatin condensation inducer in the nucleus**	**Known**	**Impaired**
**7**	***KAT7***	**Histone acetyltransferase KAT7**	**Known**	**Impaired**
Cytoskeleton				
8	*KANK2*	KN motif and ankyrin repeat domain-containing protein 2	Novel	No effect
9	*GOLGA6L10*	Golgin subfamily A member 6-like protein 10	Novel	No effect
10	*ACTB*	Actin, cytoplasmic 1	Known	No effect
11	*SUN2*	SUN domain-containing protein 2	Known	No effect
12	*TUBA1B*	Tubulin alpha-1B chain	Novel	No effect
13	*GOLGA6L10*	Golgin subfamily A member 6-like protein 10	Novel	No effect
14	*ACTG1*	Actin, cytoplasmic 2	Novel	No effect
15	*PFN1*	Profilin-1	Novel	No effect

16	*TUBB2B*	Tubulin beta-2B chain	Novel	No effect
17	*TUBA4A*	Tubulin alpha-4A chain	Novel	No effect
18	*TUBB*	Tubulin beta chain	Novel	No effect
19	*TUBB4B*	Tubulin beta-4B chain	Novel	No effect
20	*MAP7*	Ensconsin	Known	No effect
21	*TUBB4A*	Tubulin beta-4A chain	Novel	No effect
22	*TUBA1A*	Tubulin alpha-1A chain	Novel	No effect
**23**	***MTCL1***	**Microtubule cross-linking factor 1**	**Novel**	**Impaired**
24	*KRT18*	Keratin, type I cytoskeletal 18	Novel	No effect
25	*TUBB2A*	Tubulin beta-2A chain	Novel	No effect
Extracellular matrix protein				
26	*LGALS7*	Galectin-7	Novel	No effect
27	*HDGFRP2*	Hepatoma-derived growth factor-related protein 2	Known	No effect
28	*JPH1*	Junctophilin 1	Known	No effect
29	*BRPF1*	Peregrin	Known	No effect
Membrane traffic protein				
31	*SYBU*	Syntabulin	Novel	No effect
32	*LMAN1*	Lectin, Mannose Binding 1	Known	No effect
33	*LLGL1*	Lethal(2) giant larvae protein homolog 1	Novel	No effect
34	*EMD*	Emerin	Novel	No effect
35	*LEMD2*	LEM Domain Nuclear Envelope Protein 2	Known	No effect
36	*REEP4*	Receptor expression-enhancing protein 4	Known	No effect

Metabolite interconversion enzyme				
37	*LBR*	Delta(14)-sterol reductase	Known	No effect
38	*STT3B*	Dolichyl-diphosphooligosaccharide--protein glycosyltransferase subunit STT3B	Known	No effect
39	*LPL*	Lipoprotein lipase	Novel	No effect
40	*PFKP*	ATP-dependent 6-phosphofructokinase, platelet type	Novel	No effect
41	*ACSL3*	Long-chain-fatty-acid--CoA ligase 3	Novel	No effect
42	*TECR*	Very-long-chain enoyl-CoA reductase	Known	No effect
43	*TKT*	Transketolase	Known	No effect
44	*RPN1*	Dolichyl-diphosphooligosaccharide protein glycosyltransferase subunit 1	Known	No effect
45	*CPS1*	Carbamoyl-phosphate synthase [ammonia]	Novel	No effect
46	*PIK3C2A*	Phosphatidylinositol 4-phosphate 3-kinase C2 domain-containing subunit alpha	Novel	No effect
47	*DPM1*	Dolichol-phosphate mannosyltransferase subunit 1	Known	No effect
Nucleic acid binding protein				
**48**	***POLR2A***	**DNA-directed RNA polymerase;DNA-directed RNA polymerase II subunit RPB1**	**Known**	**Impaired**
49	*MSH6*	DNA mismatch repair protein Msh6	Known	No effect
**50**	***REXO1***	**RNA exonuclease 1 homolog**	**Novel**	**Impaired**
**51**	***CLASRP***	**CLK4-associating serine/arginine rich protein**	**Novel**	**Impaired**
**52**	***TCOF1***	**Treacle protein**	**Known**	**Impaired**
53	*PAPD7*	Terminal nucleotidyltransferase 4A	Novel	No effect
54	*DHX38*	Pre-mRNA-splicing factor ATP-dependent RNA helicase PRP16	Novel	No effect
55	*LSM14B*	Protein LSM14 homolog B	Novel	No effect
56	*DHX16*	Pre-mRNA-splicing factor ATP-dependent RNA helicase DHX16	Known	No effect

57	*NOP58*	Nucleolar protein 58	Known	No effect
58	*FAM133A*	Protein FAM133A	Novel	No effect
**59**	***DROSHA***	**Ribonuclease 3**	**Known**	**Impaired**
60	*SRRM1*	Serine/arginine repetitive matrix protein 1	Known	No effect
61	*SUB1*	Activated RNA polymerase II transcriptional coactivator p15	Known	No effect
62	*COIL*	Coilin	Known	No effect
63	*RNPS1*	RNA-binding protein with serine-rich domain 1	Known	No effect
64	*FAM133B*	Protein FAM133B	Novel	No effect
**65**	***GPATCH2***	**G patch domain-containing protein 2**	**Known**	**Impaired**
66	*MCM7*	DNA replication licensing factor MCM7	Known	No effect
67	*CCNL1*	DNA replication licensing factor MCM2	Known	No effect
68	*CTR9*	RNA polymerase-associated protein CTR9 homolog	Known	No effect
69	*ZRANB2*	Zinc finger Ran-binding domain-containing protein 2	Known	No effect
**70**	***THRAP3***	**Thyroid hormone receptor-associated protein 3**	**Known**	**Impaired**
71	*H2AFV*	Histone H2A.V	Novel	No effect
**72**	***BCLAF1***	**Bcl-2-associated transcription factor 1**	**Known**	**Impaired**
73	*RBM39*	RNA-binding protein 39	Novel	No effect
74	*PRPF38A*	Pre-mRNA-splicing factor 38A	Novel	No effect
**75**	***IWS1***	**Protein IWS1 homolog**	**Known**	**Impaired**
76	*CWC25*	Pre-mRNA-splicing factor CWC25 homolog	Novel	No effect
77	*RSRC1*	Serine/Arginine-related protein 53	Novel	No effect
78	*MMS22L*	Protein MMS22-like	Novel	No effect

**79**	***GPBP1***	**Vasculin**	**Novel**	**Impaired**
80	*HLTF*	Helicase-like transcription factor	Novel	No effect
81	*SSRP1*	FACT complex subunit SSRP1	Novel	No effect
82	*SNAPC4*	snRNA-activating protein complex subunit 4	Novel	No effect
83	*ERCC5*	DNA repair protein complementing XP-G cells	Novel	No effect
84	*RAD50*	DNA repair protein RAD50	Novel	No effect
85	*AKAP17A*	A-kinase anchor protein 17A	Novel	No effect
**86**	***SRRM2***	**Serine/arginine repetitive matrix protein 2**	**Known**	**Impaired**
87	*ATRX*	Transcriptional regulator ATRX	Novel	No effect
88	*PAF1*	RNA polymerase II-associated factor 1 homolog	Novel	No effect
89	*POLD3*	DNA polymerase delta subunit 3	Novel	No effect
90	*PRPF19*	Pre-mRNA-processing factor 19	Novel	No effect
91	*CBX2*	Chromobox 2	Known	No effect
92	*RTF1*	RNA polymerase-associated protein RTF1 homolog	Novel	No effect
93	*PCNA*	Proliferating cell nuclear antigen	Known	No effect
Protein modifying enzymes				
94	*CDK11A*	Cyclin-dependent kinase 11A	Known	No effect
95	*CDC42BPA*	Serine/threonine-protein kinase MRCK alpha	Novel	No effect
96	*TOPORS*	E3 ubiquitin-protein ligase Topors	Novel	No effect
**97**	***RBBP6***	**E3 ubiquitin-protein ligase RBBP6**	**Known**	**Impaired**
98	*RNF111*	E3 ubiquitin-protein ligase Arkadia	Novel	No effect
99	*TFRC*	Transferrin receptor protein 1	Novel	No effect

100	*ARL6IP4*	ADP-ribosylation factor-like protein 6-interacting protein 4	Novel	No effect
101	*PHF8*	Histone lysine demethylase PHF8	Novel	No effect
102	*FBXL19*	F-box/LRR-repeat protein 19	Novel	No effect
103	*SCD*	Acyl-CoA desaturase	Novel	No effect
**104**	***SETD2***	**Histone-lysine N-methyltransferase SETD2**	**Known**	**Impaired**
105	*SGPL1*	Sphingosine-1-phosphate lyase 1	Novel	No effect
106	*CDK11B*	Cyclin-dependent kinase 11B	Novel	No effect
Protein binding activity modulators				
107	*RAN*	GTP-binding nuclear protein Ran	Known	No effect
108	*CCNK*	Cyclin-K	Known	No effect
109	*CCNL2*	Cyclin-L2	Known	No effect
110	*ARF3*	ADP-ribosylation factor 3	Known	No effect
111	*ARF1*	ADP-ribosylation factor 1	Known	No effect
112	*CCNL1*	Cyclin-L1	Novel	No effect
113	*ARF4*	ADP-ribosylation factor 4	Known	No effect
114	*NKAP*	NF-kappa-B-activating protein	Novel	No effect
115	*UBB*	Polyubiquitin-B	Novel	No effect
Scaffold adaptor proteins				
116	*CHD2*	Chromodomain-helicase-DNA-binding protein 2	Known	No effect
**117**	***DVL2***	**Segment polarity protein dishevelled homolog DVL-2**	**Known**	**Impaired**
**118**	***PRICKLE3***	**Prickle planar cell polarity protein 3**	**Novel**	**Impaired**
**119**	***BRD2***	**Bromodomain-containing protein 2**	**Known**	**Impaired**

120	*CACTIN*	Cactin	Novel	No effect
**121**	***DVL1P1***	**putative segment polarity protein dishevelled homolog DVL1P1**	**Novel**	**Impaired**
**122**	***DVL1***	**Segment polarity protein dishevelled homolog DVL-1**	**Known**	**Impaired**
**123**	***LENG8***	**Leukocyte receptor cluster member 8**	**Known**	**Impaired**
124	*CEP170*	Centrosomal protein of 170 kDa	Known	No effect
**125**	***DLG5***	**Disks large homolog 5**	**Novel**	**Impaired**
**126**	***DVL3***	**Segment polarity protein dishevelled homolog DVL-3**	**Known**	**Impaired**
Transporter proteins				
127	*OXA1L*	Mitochondrial inner membrane protein OXA1L	Novel	No effect
128	*SLC27A4*	Long-chain fatty acid transport protein 4	Novel	No effect
129	*POM121*	Nuclear envelope pore membrane protein POM 121	Novel	No effect
130	*FAM120A*	Constitutive coactivator of PPAR-gamma-like protein 1	Novel	No effect
131	*SLC16A3*	Monocarboxylate transporter 4	Novel	No effect
132	*SLC1A5*	Neutral amino acid transporter B(0)	Known	No effect
133	*POM121C*	Nuclear envelope pore membrane protein POM 121C	Novel	No effect
134	*SLC16A1*	Monocarboxylate transporter 1	Novel	No effect
135	*SLC25A3*	Phosphate carrier protein, mitochondrial	Novel	No effect
**136**	***SLC25A5***	**ADP/ATP translocase 2**	**Known**	**Impaired**
137	*SLC2A1*	Solute carrier family 2, facilitated glucose transporter member 1	Novel	No effect
138	*SLC7A5*	Large neutral amino acids transporter small subunit 1	Novel	No effect
Translational protein				
139	*RPL13*	60S ribosomal protein L13	Known	No effect

140	*RPL11*	60S ribosomal protein L11	Novel	No effect
141	*RPS3*	40S ribosomal protein S3	Novel	No effect
142	*RPS18*	40S ribosomal protein S18	Known	No effect
143	*TUFM*	Elongation factor Tu, mitochondrial	Novel	No effect
144	*RPS27*	40S ribosomal protein S27	Novel	No effect
145	*BOP1*	Ribosome biogenesis protein BOP1	Known	No effect
146	*RPL10L*	60S ribosomal protein L10-like	Novel	No effect
147	*RPS11*	40S ribosomal protein S11	Novel	No effect
148	*PELO*	Protein pelota homolog	Known	No effect
149	*RPS2*	40S ribosomal protein S2	Novel	No effect
Dead box proteins				
150	*DDX20*	Probable ATP-dependent RNA helicase DDX20	Novel	No effect
151	*DDX28*	Probable ATP-dependent RNA helicase DDX28	Novel	No effect
152	*DDX39A*	ATP-dependent RNA helicase DDX39A	Novel	No effect
**153**	***DDX46***	**Probable ATP-dependent RNA helicase DDX46**	**Known**	**Impaired**
**154**	***DDX47***	**Probable ATP-dependent RNA helicase DDX47**	**Known**	**Impaired**
**155**	***DDX5***	**Probable ATP-dependent RNA helicase DDX5**	**Known**	**Impaired**
Ribosomal proteins				
156	*RP9*	Retinitis pigmentosa 9 protein	Novel	No effect
157	*RPS16*	40S ribosomal protein S16	Novel	No effect
158	*RPS27A*	Ubiquitin-40S ribosomal protein S27a	Novel	No effect
159	*RPS6KA1*	Ribosomal protein S6 kinase alpha-1	Novel	No effect

160	*RPS6KA3*	Ribosomal protein S6 kinase alpha-3	Novel	No effect
161	*RPS6KA4*	Ribosomal protein S6 kinase alpha-4	Novel	No effect
162	*WDR12*	Ribosome biogenesis protein WDR12	Novel	No effect
163	*NOLC1*	Nucleolar and coiled-body phosphoprotein	Novel	No effect
Miscellaneous				
**164**	***RICTOR***	**Rapamycin-insensitive companion of mTOR**	**Novel**	**Impaired**
165	*PPIG*	Peptidyl-prolyl cis-trans isomerase G	Novel	No effect
**166**	***GPATCH8***	**G patch domain-containing protein 8**	**Novel**	**Impaired**
167	*EPB41L4A*	Band 4.1-like protein 4A	Novel	No effect
**168**	***BRD3***	**Bromodomain-containing protein 2/3**	**Known**	**Impaired**
169	*ESYT2*	Extended synaptotagmin-2	Novel	No effect
170	*DNAJA3*	DnaJ homolog subfamily A member 3, mitochondrial	Novel	No effect
171	*C12ORF43*	Protein CUSTOS	Novel	No effect
172	*SDAD1*	Protein SDA1 homolog	Novel	No effect
173	*FARP1*	FERM, ARHGEF and pleckstrin domain-containing protein 1	Novel	No effect
**174**	***CFAP20***	**Cilia- and flagella-associated protein 20**	**Novel**	**Impaired**
175	*ZNF90*	Zinc finger protein 90	Novel	No effect
176	*ANKS3*	Ankyrin repeat and SAM domain-containing protein 3	Novel	No effect
**177**	***ZC3H18***	**Zinc finger CCCH domain-containing protein 18**	**Known**	**Impaired**
178	*MCTP2*	Multiple C2 and transmembrane domain-containing protein 2	Novel	No effect
179	*LUC7L3*	Luc7-like protein 3	Novel	No effect
**180**	***GRAMD1A***	**GRAM Domain Containing 1A**	**Novel**	**Impaired**

181	*TONSL*	Tonsoku-like protein	Novel	No effect
182	*HSPD1*	60 kDa heat shock protein, mitochondrial	Novel	No effect
183	*KRI1*	Protein KRI1 homolog	Novel	No effect
184	*TTC14*	Tetratricopeptide repeat protein 14	Novel	No effect
185	*PAF1*	Peroxisome biogenesis factor 2	Novel	No effect
186	*SREK1IP1*	Protein SREK1IP1	Novel	No effect
**187**	***KIAA1522***	**Uncharacterized protein KIAA1522**	**Known**	**Impaired**
188	*ATAD3A*	ATPase family AAA domain-containing protein 3A	Novel	No effect
189	*SMN1/SMN2*	Survival motor neuron protein	Novel	No effect
190	*PHRF1*	PHD and RING finger domain-containing protein 1	Novel	No effect
191	*NKAPL*	NKAP-like protein	Novel	No effect
**192**	***ANKRD11***	**Ankyrin repeat domain-containing**	**Novel**	**Impaired**
193	*C18ORF25*	Uncharacterized protein C18orf25	Novel	No effect
**194**	***CFAP97***	**Cilia- and flagella-associated protein 97**	**Novel**	**Impaired**

Note: Bold are the proteins showing impaired interaction, green color is for proteins showing impaired interaction but already known partners of CK2 whereas yellow colored proteins are novel showing impaired interaction.

Table S4. List of differentially expressed genes found in transcriptome data

Table S5. List of phosphoproteome found in wild-type and patient LCLs

Table S6. List of nonphosphorylated motifs observed in patient LCLs

Table S7. List of hyper-phosphorlyted motifs observed in patient LCLs

Table S8. Putative CK2 traget motifs found non-phosphorylated in the patient LCLs

Table S9. List of the reported proteins containing CK2 phosphosites, hypo-phosphorylated in mutant LCLs.

Serial No.	Unique identifier	Gene name	Protein name	-Log T-test p-value Mut_WT	-Log T-test difference Mut_WT	Mean log2 Mut	Mean log2 Mut	Position	A.A	Motif sequence
1	UID878	*MATR3*	A0A0R4J2E8	1,84661	-2,90348	0	25,3679	604	S	KDKSRKRSYSPDGKESPSDKKSKTDGSQKTE
2	UID1267	*SRRM1*	A9Z1X7	2,24326	-3,37243	0	25,9	724	S	VRRGASSSPQRRQSPSPSTRPIRRVSRTPEP
3	UID2107	*TCOF1*	J3KQ96	3,16556	-3,70411	0	26,4862	349	S	KPEEDSESSSEESSDSEEETPAAKALLQAKA
4	UID2831	*HNRNPA1*	F8W6I7	1,67231	-1,38036	0	24,3194	298	S	FAKPRNQGGYGGSSSSSSYGSGRRF______
5	UID3058	*CEP170*	H0Y2V6	1,85526	-2,24501	0	24,2167	922	S	LVTGETERKSTQKRKSFTSLYKDRCSTGSPS
6	UID3358	*PML*	H3BT57	1,72813	-2,80895	0	25,1201	38	S	MPPPETPSEGRQPSPSPSPTERAPASEEEFQ
7	UID5310	*CTPS1*	P17812	3,32585	-8,23113	0	30,665	562	S	SVGRLSHYLQKGCRLSPRDTYSDRSGSSSPD
8	UID5428	*LMNB1*	P20700	2,48576	-0,919029	0	23,3732	278	S	ELEQTYHAKLENARLSSEMNTSTVNSAREEL
9	UID5578	*RPL13*	P26373	1,36496	-1,8067	0	23,8933	106	S	ARTIGISVDPRRRNKSTESLQANVQRLKEYR
10	UID5814	*DEK*	P35659	1,96599	-1,40587	0	24,0236	231	S	KAKRTKCPEILSDESSSDEDEKKNKEESSDD
11	UID5846	*RBMX*	P38159	1,47344	-1,66209	0	24,2072	221	S	YLSPRDDGYSTKDSYSSRDYPSSRDTRDYAP
12	UID5867	*BRCA1*	P38398	3,5814	-2,36493	0	24,7581	1642	S	AMEESVSREKPELTASTERVNKRMSMVVSGL
13	UID6696	*SPTBN1*	Q01082	1,60399	-1,43035	0	23,9776	825	S	LPQEHAESPDVRGRLSGIEERYKEVAELTRL
14	UID6945	*SSRP1*	Q08945	1,56733	-1,97234	0	24,6804	659	S	SRGSSSKSSSRQLSESFKSKEFVSSDESSSG
15	UID7394	*SQSTM1*	Q13501	2,01105	-2,56307	0	24,852	266	S	LGIEVDIDVEHGGKRSRLTPVSPESSSTEEK
16	UID7746	*GAPVD1*	Q14C86	1,74977	-2,42111	0	24,8049	914	S	RSRSSDIVSSVRRPMSDPSWNRRPGNEEREL
17	UID7874	*SKIV2L*	Q15477	1,51594	-1,923	0	24,4506	245	S	AVGQPGGPRGDTVSASPCSAPLARASSLEDL

18	UID8348	*ZC3H13*	Q5T200	1,85281	-1,75648	0	24,1883	325	S	KRDKPRSTSPAGQHHSPISSRHHSSSSQSGS
19	UID8683	*ARL6IP4*	Q66PJ3	3,53654	-2,2089	0	25,173	239	S	TSQGRKASTAPGAEASPSPCITERSKQKARR
20	UID10997	*IWS1*	Q96ST2	1,9635	-1,80155	0	24,6343	261	S	RISDSESEDPPRHQASDSENEELPKPRISDS
21	UID11340	*HIRIP3*	Q9BW71	2,11588	-2,53742	0	24,6738	305	S	DSEEEQKEAASSGDDSGRDREPPVQRKSEDR
22	UID11547	*KLC2*	Q9H0B6	2,47137	-2,56392	0	24,6784	507	S	VELLKDGSGRRGDRRSSRDMAGGAGPRSESD
23	UID12778	*SRRM2*	Q9UQ35	3,20399	-5,75498	0	28,101	353	S	KDKDKKEKSATRPSPSPERSSTGPEPPAPTP
24	UID13128	*THRAP3*	Q9Y2W1	2,32333	-2,48784	0	24,7002	560	S	RDKLGAKGDFPTGKSSFSITREAQVNVRMDS
25	UID14242	*ANP32A*	P39687	2,42683	-5,44456	0	28,025	15	T	_MEMGRRIHLELRNRTPSDVKELVLDNSRSN
26	UID14552	*IFI16*	Q16666	1,964	-1,70463	0	24,2853	779	T	RKNKKDILNPDSSMETSPDFFF_________
27	UID26065	*CHAMP1*	Q96JM3	1,9457	-2,00867	0	24,7963	386	S	KSSSVSPSSWKSPPASPESWKSGPPELRKTA
28	UID12196	*FAM120A*	Q9NZB2	2,50489	-1,5054	0	24,4097	1023	S	YKNQAAIQGRPPYAASAEEVAKELKSKSGES
29	UID12507	*FAM208A*	Q9UK61	3,65328	-2,67363	0	25,5076	673	S	SRGEAIISGKQRSSHSLDYDKDRVKELINLI
30	UID11394	*FANCD2*	Q9BXW9	2,68882	-1,90693	0	24,5454	891	S	GSKTSSSDTLSEEKNSECDPTPSHRGQLNKE
31	UID12086	*GPATCH2*	Q9NW75	2,09749	-1,82992	0	24,4595	54	S	SSEQARGGFAETGDHSRSISCPLKRQARKRR
32	UID24474	*HDGFRP2*	Q7Z4V5	2,08159	-2,05645	0	24,5757	369	S	RERADRGEAERGSGGSSGDELREDDEPVKKR
33	UID7122	*ILF3*	Q12906	4,37204	-2,47526	0	24,8753	810	S	GGGGSDYNYESKFNYSGSGGRSGGNSYGSGG
34	UID25303	*KDM2B*	Q8NHM5	2,00112	-3,10998	0	25,4933	474	S	KKPKAPALRFLKRTLSNESEESVKSTTLAVD
35	UID4291	*KIF1C*	O43896	4,14194	-5,04269	0	27,4672	1092	S	RYPPYTTPPRMRRQRSAPDLKESGAAV____
36	UID28523	*PAXBP1*	Q9Y5B6	1,42533	-3,80199	0	26,7574	557	S	AREQTGKMADHLEGLSSDDEETSTDITNFNL

37	UID24917	*PELP1*	Q8IZL8	2,17346	-3,27119	0	26,0756	485	S	ADALKLRSPRGSPDGSLQTGKPSAPKKLKLD
38	UID7149	*PRDM2*	Q13029	1,75865	-1,59707	0	24,4618	739	S	SMLPVTSSRFKRRTSSPPSSPQHSPALRDFG
39	UID9258	*RBBP6*	Q7Z6E9	3,86433	-2,66185	0	25,311	1699	S	QVGISRNQSHSSPSVSPSRSHSPSGSQTRSH
40	UID6526	*RPS3A*	P61247	2,09668	-2,08137	0	24,5827	236	S	PKFELGKLMELHGEGSSSGKATGDETGAKVE
41	UID18056	*SCAF11*	F8VXG7	3,02145	-1,931	0	24,9008	688	S	RSPKRDTTRESRRSESLSPRRETSRENKRSQ
42	UID1773	*SRRT*	C9JUL9	2,12054	-1,95044	0	24,6615	11	S	_____MAPSDRAMGDSDDEYDRRRRDKFRRE
43	UID6864	*SRSF11*	Q05519	4,17754	-9,0552	0	31,9729	449	S	SEKEKKEEKKPIETGSPKTKECSVEKGTGDS
44	UID8408	*UBR4*	Q5T4S7	3,89185	-3,93232	0	26,2725	1760	S	FQSEPRISESLVRHASTSSPADKAKVTISDG
45	UID1568	*USP39*	B9A018	2,45224	-2,63278	0	25,3719	43	S	VKRERDREREPEAASSRGSPVRVKREFEPAS
46	UID11959	*ANLN*	Q9NQW6	2,4176	-3,46981	0	26,1905	65	S	QQPLSGGEEKSCTKPSPSKKRCSDNTEVEVS
47	UID17174	*CLASP2*	E3W994	2,82457	-3,43792	0	25,6775	529	S	ARSSRIPRPSVSQGCSREASRESSRDTSPVR
48	UID27144	*CWC22*	Q9HCG8	3,27482	-1,9837	0	24,9613	61	S	FDYSRSDYEHSRRGRSYDSSMESRNRDREKR
49	UID8000	*DBN1*	Q16643	2,52321	-2,23062	0	24,9901	339	S	SHRRMAPTPIPTRSPSDSSTASTPVAEQIER
50	UID4122	*FYB*	O15117	2,69347	-2,25251	0	25,244	209	S	PKPAFGQKPPLSTENSHEDESPMKNVSSSKG
51	UID24622	*KTN1*	Q86UP2	1,9361	-2,04979	0	24,7763	75	S	KKNKKKEIQNGNLHESDSESVPRDFKLSDAL
52	UID2447	*LMO7*	E9PMS6	4,33436	-4,71109	0	27,2906	316	S	QKWQDDLAKWKDRRKSYTSDLQKKKEEREEI
53	UID9371	*LUZP1*	Q86V48	2,76122	-3,51584	0	25,8246	440	S	SFTNRRAAKASHMGVSTDSGTQETKKTEDRF
54	UID7357	*MYO9B*	Q13459	2,86149	-3,56847	0	26,7478	1972	S	GDEDREKEILIERIQSIKEEKEDITYRLPEL
55	UID9136	*SETX*	Q7Z333	2,15569	-1,95276	0	24,2906	642	S	MGKTSRKDMHCLEASSPTFSKEPMKVQDSVL

56	UID981	*TJP2*	A0A1B0GTW1	1,37164	-2,40028	0	25,0543	229	S	YSERSRLNSHGGRSRSWEDSPERGRPHERAR
57	UID6983	*AHNAK*	Q09666	1,75803	-2,2796	0	25,4353	5731	S	KFNFSKPKGKGGVTGSPEASISGSKGDLKSS
58	UID21347	*ATRX*	P46100	2,21981	-2,68854	0	25,3956	675	S	KTTPLRRPTETNPVTSNSDEECNETVKEKQK
59	UID6628	*BASP1*	P80723	3,10991	-4,6497	0	27,2906	194	S	AAPSSKETPAATEAPSSTPKAQGPAASAEEP
60	UID2679	*CCDC82*	F5H777	1,53395	-2,06881	0	24,9692	219	S	KVGVKRPRRVVEDEGSSVEMEQKTPEKTLAA
61	UID14383	*CENPC*	Q03188	4,39618	-2,24372	0	25,1936	130	T	EVHQKILATDVSSKNTPDSKKISSRNINDHH
62	UID902	*DENND4A*	A0A0U1RR27	2,22261	-2,58959	0	24,7865	966	S	GYNSLSKDEVRRGDTSTEDIQEEKDKKGSDC
63	UID8794	*DHX57*	Q6P158	3,56005	-2,8242	0	25,112	74	S	DDGDDFCIFSESRRPSRPSNSNISKGESRPK
64	UID29344	*EIF3G*	O75821	1,43218	-1,71542	0	23,6488	41	T	VTSELLKGIPLATGDTSPEPELLPGAPLPPP
65	UID28941	*HMX3*	A6NHT5	2,0436	-2,70737	0	25,8685	149	T	ASEKALLRDSSPASGTDRDSPEPLLKADPDH
66	UID13062	*INPP5F*	Q9Y2H2	1,77021	-1,20438	0	24,2196	907	S	CGIIASAPRLGSRSQSLSSTDSSVHAPSEIT
67	UID7325	*PPIG*	Q13427	1,77154	-2,08697	0	25,283	745	S	NDHVHEKNKKFDHESSPGTDEDKSG______
68	UID501	*RRBP1*	A0A0A0MRV0	2,5356	-1,67658	0	24,3632	1277	S	KSHVEDGDIAGAPASSPEAPPAEQDPVQLKT
69	UID7670	*RRP1B*	Q14684	1,82784	-2,40115	0	24,3473	513	S	SQSGPSGSHPQGPRGSPTGGAQLLKRKRKLG
70	UID5331	*SON*	P18583	2,0521	-1,5906	0	24,263	2129	S	QSKEDDDVIVNKPHVSDEEEEEPPFYHHPFK
71	UID8638	*ZMYM4*	Q5VZL5	2,88464	-1,65957	0	24,0498	1256	S	SPRSDPLGSTQDHALSQESSEPGCRVRSIKL
72	UID785	*MEF2C*	A0A0D9SGI5	2,09559	-2,85107	0	25,218	419	S	TPSRYPQHTRHEAGRSPVDSLSSCSSSYDGS

1 Note Mut = Mutant_p.Asp32His, WT = Wild type, A.A = amino acid.

Supplemental Method

Variants identification

Patient 1

To reveal the disease-causing DNA variant(s), we subjected trio — patient, both parents — of family 1 to whole-exome sequencing using NimbleGen SeqCap EZ Human Exome Library v2.0 kit. Samples were run on an Illumina HiSeq 2000 system using a paired-end 2 × 100 bp protocol and data was generated as detailed previously.[3; 4] For filtering and prioritizing genetic variants, we used our in-house VARBANK dataset and analysis tool kit.[5] Method of exome sequencing in remaining individuals is given in the supplementary section.

Patient 2

Trio clinical exome sequencing was performed for the Patient 2 and her parents by GeneDx (Gaithersburg, MD) using a proprietary capture system developed by GeneDx for next generation sequencing with CNV calling. Sequence was aligned to the UCSC build hg19 reference sequence. Mean depth of coverage was 114X with quality threshold (>10X reads) of 98.6% of exome. GeneDx's XomeAnalyzer was used to analyzed variant between the probands, parents and reference.

Patient 3

Genetic evaluation for patient 3 began with simultaneous chromosome analysis and chromosomal microarray. Chromosome analysis demonstrated a normal female karyotype. Chromosomal microarray via whole-genome array CGH and genotype with 180,000 oligonucleotide probes on the GRCh37/UCSC hg 19 build did not identify any copy number variations. Exome sequencing through a commercial laboratory identified a heterozygous pathogenic variant in *CSNK2B*, specified c.94G>A (p.Asp32Asn) (NM_001320.7). Parental samples were not submitted to determine if this *CSKN2B* pathogenic variant was inherited or *de novo*, though parents have typical intellect and no contributory medical history. No other pathogenic variants or variants of uncertain significance were reported for the patient on exome sequencing.

Patient 4

To reveal the pathogenic variant in patient 4, we employed chromosomal microarray analysis which showed normal number of chromosomes. In parallel, this patient was also found negative for pathogenic mutations in *SCN1A* and *FMR1,* later is a causative gene of fragile X syndrome.[6] Eventually, WES revealed a novel pathogenic *de novo*

variant NM_001320.7:c.374C>G;p.Ser125* in *CSNK2B*.

Patient 5

Initailly, we employed targeted sequencing of genes associated with seizures/epilepsy which did not show any pathogenic variant, so the patient underwent singleton exome sequencing that identified the *CSNK2B* variant — NM_001320.7:c.367+5delG. Inheritance of the variant is unknown.

Extended information for GestaltMatcher analysis

GestaltMatcher spanned a 320-dimensional clinical face phenotype space (CFPS) defined by the feature vectors derived from DeepGestalt.[7] Each image is a point located in CFPS, and the cosine distance quantified the similarity between two images in the space. In CFPS, we consider the images with close distance to have a high overlap of syndromic facial features. Therefore, we ranked the patients by sorting the cosine distance.

We performed pairwise comparisons on the seven photos of patient-1 to patient-7 along with 3,533 images from 2,516 diagnosed patients with 816 syndromes in the Face2Gene database. The following two criteria selected the 2,571 patients from the database. A patient's diagnosed syndrome was not included in the model training and had less than seven subjects. By these selection criteria, we formed the CFPS with syndromes that have not been seen by the model and have very few subjects simulating the ultra-rare diseases.

Copy number analysis

250 ng of DNA were digested with NspI and amplified with ligation-mediated PCR, purified, fragmented with DNase I, labelled with biotin and hybridized on array CytoScan HD (Thermo Fisher Scientific). After washing and staining with streptavidin on a GeneChip Fluidics Station 450, the arrays were scanned by GeneChip Scanner 3000. Finally, CEL files were generated with GeneChip Command Console software (Thermo Fisher Scientific) and analysed with the programs ChAS 3.0 (Thermo Fisher Scientific) and Nexus Copy Number 7.5 (BioDiscovery).

Minigene construction, transfection and RT-PCR

To reveal the consequences of NM_001320.7:c.367+5delG on *CSNK2B* transcript splicing, we constructed minigene vectors with the help of GenScript Biotech (Netherlands) B.V.. For this purpose, genomic fragments of wild-type and mutant *CSNK2B* (ENST00000375882.7;CSNK2B-203) spaning exon 4 to exon 6 were cloned

in mammalian expression vector, pCMV-3Tag-3a, using SacI/XhoI restriction sites. We used 1mg/ml polyethylenimine (PEI, Polysciences, 23966) to transfect HeLa cells for 24 hours. Subsequently, cells were subjected to isolate total RNA with the help of RNeasy Mini kit (Qiagen, 74104) which later was converted into cDNA using SuperScript III reverse transcriptase (RT) enzyme (ThermoFisher Scintific, 18080093). For PCR amplification, we used vector and insert specific forward and reverse primers, respectively. Oligonucleotides sequences are listed in Table S2. To detect different transcripts and aberrant splicing, amplified PCR product was subjected for Sanger sequencing.

Supplementary References

1. Savatt, J.M., Azzariti, D.R., Faucett, W.A., Harrison, S., Hart, J., Kattman, B., Landrum, M.J., Ledbetter, D.H., Miller, V.R., and Palen, E. (2018). ClinGen's GenomeConnect registry enables patient-centered data sharing. Human mutation 39, 1668-1676.
2. Ernst, M.E., Baugh, E.H., Thomas, A., Bier, L., Lippa, N., Stong, N., Mulhern, M.S., Kushary, S., Akman, C.I., Heinzen, E.L., et al. (2021). CSNK2B: A broad spectrum of neurodevelopmental disability and epilepsy severity. Epilepsia.
3. Hussain, M.S., Baig, S.M., Neumann, S., Nurnberg, G., Farooq, M., Ahmad, I., Alef, T., Hennies, H.C., Technau, M., Altmuller, J., et al. (2012). A truncating mutation of CEP135 causes primary microcephaly and disturbed centrosomal function. Am J Hum Genet 90, 871-878.
4. Hussain, M.S., Baig, S.M., Neumann, S., Peche, V.S., Szczepanski, S., Nurnberg, G., Tariq, M., Jameel, M., Khan, T.N., Fatima, A., et al. (2013). CDK6 associates with the centrosome during mitosis and is mutated in a large Pakistani family with primary microcephaly. Human Molecular Genetics 22, 5199-5214.
5. Hussain, M.S., Battaglia, A., Szczepanski, S., Kaygusuz, E., Toliat, M.R., Sakakibara, S., Altmuller, J., Thiele, H., Nurnberg, G., Moosa, S., et al. (2014). Mutations in CKAP2L, the Human Homolog of the Mouse Radmis Gene, Cause Filippi Syndrome. Am J Hum Genet 95, 622-632.
6. Siomi, H., Choi, M., Siomi, M.C., Nussbaum, R.L., and Dreyfuss, G. (1994). Essential role for KH domains in RNA binding: impaired RNA binding by a mutation in the KH domain of FMR1 that causes fragile X syndrome. Cell 77, 33-39.
7. Gurovich, Y., Hanani, Y., Bar, O., Nadav, G., Fleischer, N., Gelbman, D., Basel-Salmon, L., Krawitz, P.M., Kamphausen, S.B., Zenker, M., et al. (2019). Identifying facial phenotypes of genetic disorders using deep learning. Nat Med 25, 60-64.
